# Anti-Cholera toxin activity of selected polyphenols from *Careya arborea*, *Punica granatum*, and *Psidium guajava*

**DOI:** 10.3389/fcimb.2023.1106293

**Published:** 2023-04-11

**Authors:** Rajitha Charla, Priyanka P. Patil, Vishal S. Patil, Vishwambhar V. Bhandare, Veeresh Karoshi, Venkanna Balaganur, Rajesh K. Joshi, Darasaguppe R. Harish, Subarna Roy

**Affiliations:** ^1^ Indian Council of Medical Research - National Institute of Traditional Medicine, Belagavi, Karnataka, India; ^2^ KLE Academy of Higher Education and Research (KAHER), Belagavi, India; ^3^ Department of Microbiology, Shivaji University, Kolhapur, India; ^4^ Indian Council of Agricultural Research – Krishi Vigyan Kendra, Bagalkot, Karnataka, India; ^5^ University of Agricultural Sciences, Dharwad, Karnataka, India

**Keywords:** cell free culture filtrate, Cholera toxin, cytotoxicity, docking, GM1 ELISA, molecular dynamics simulations

## Abstract

**Introduction:**

*Careya arborea*, *Punica granatum*, and *Psidium guajava* are traditionally used to treat diarrheal diseases in India and were reported to show anti-Cholera toxin activity from our earlier studies. As polyphenols are reported to neutralize Cholera toxin (CT), the present study investigated the inhibitory activity of selected polyphenols from these plants against CTB binding to GM1 receptor using *in silico*, *in vitro*, and *in vivo* approaches.

**Methods:**

Molecular modelling approach was used to investigate the intermolecular interactions of selected 20 polyphenolic compounds from three plants with CT using DOCK6. Based on intermolecular interactions, two phenolic acids, Ellagic acid (EA) and Chlorogenic acid (CHL); two flavonoids, Rutin (RTN) and Phloridzin (PHD) were selected along with their respective standards, Gallic acid (GA) and Quercetrin (QRTN). The stability of docked complexes was corroborated using molecular dynamics simulation. Furthermore, in vitro inhibitory activity of six compounds against CT was assessed using GM1 ELISA and cAMP assay. EA and CHL that showed prominent activity against CT in *in vitro* assays were investigated for their neutralizing activity against CT-induced fluid accumulation and histopathological changes in adult mouse.

**Results and discussion:**

The molecular modelling study revealed significant structural stability of the CT-EA, CT-CHL, and CT-PHD complexes compared to their respective controls. All the selected six compounds significantly reduced CT-induced cAMP levels, whereas EA, CHL, and PHD exhibited > 50% binding inhibition of CT to GM1. The EA and CHL that showed prominent neutralization activity against CT from *in vitro* studies, also significantly decreased CT-induced fluid accumulation and histopathological changes in adult mouse. Our study identified bioactive compounds from these three plants against CT-induced diarrhea.

## 1 Introduction

Cholera toxin (CT), a heterohexameric protein released extracellularly by *Vibrio cholerae* in soluble form, is the key virulence factor responsible for diarrhea observed in cholera, which remains the major global health burden, especially in countries with poor sanitation strategies (World Health Organization, 2019). CT belongs to the larger family of AB toxins characterized by having an enzymatically active A-domain responsible for inducing toxicity and a cell-binding B-domain responsible for cell entry (Merritt and Hol, 1995). An A-subunit is comprised of an enzymatically active A1-chain which is non-covalently linked to the pentameric ring of B-subunits *via* the A2-chain. CT binds to ganglioside GM1 located on the outer leaflet of apical membranes of intestinal epithelial cells (Pang et al., 2004) followed by endocytosis and then traffics retrograde from the plasma membrane to the trans-golgi network, eventually reaching the endoplasmic reticulum (ER) (Wolf et al., 2002). The A2-chain has a KDEL (Lysine, Aspartic acid, Glutamic acid, and Leucine) sequence at its C-terminus which is responsible for retrograde transport of the toxin to ER (Chinnapen et al., 2007).

Inside the ER, the A-subunit dissociates from the B pentamer, followed by cleavage between A1 and A2 polypeptide chains. Protein disulfide isomerase (PDI) binds to and unfolds A1-chain, which is retro-translocated through the Sec61p channel from the ER lumen, exploiting the ER-associated protein degradation pathway (ERAD) (Taylor et al., 2011). A1-chain escapes Ubiquitin-mediated protein degradation due to its low lysine content and finally enters the cytosol (Hazes and Read, 1997). In the cytosol, the A1 chain catalyses the transfer of ADP-ribose from NAD to G protein G_sα_ resulting in permanent ADP-ribosylation of G_sα_ and activation of adenylate cyclase, which continuously stimulates the production of cyclic AMP (Cassel and Pfeuffer, 1978). The persistent elevation in cAMP levels activates the cystic fibrosis transmembrane conductance regulator (CFTR), causing a dramatic efflux of ions and water from infected enterocytes, leading to diarrhea. Though CT intoxication involves a cascade of events, the initial step in toxin internalization is its binding to the GM1 receptor on epithelial cells, which serves as a desirable target for drug discovery, thus blocking its entry into enterocytes.

Despite CT being responsible for the pathogenesis observed in cholera, treatment regimen involves oral and intravenous rehydration therapy along with the use of antibiotics such as tetracycline and azithromycin targeting the bacteria (Leibovici-Weissman et al., 2014). Over the years, emergence of antimicrobial resistance in *V. cholerae* majorly contributed by acquisition of extrachromosomal mobile genetic elements from related bacterial species (Das et al., 2020) is eventually leading to repeated outbreaks of cholera worldwide (Dengo-Baloi et al., 2012). Therefore, a number of phytocompounds have been investigated for anti-cholera activity. The potency of phytocompounds against CT has been demonstrated either by disruption of the binding of CTB to GM1, subsequently preventing its internalization or by inhibition of CTA1 catalysed ADP-ribosylation of G_sα._ A comprehensive analysis of available scientific literature revealed that most of the phytocompounds reported against CT are polyphenols. The term “polyphenols” refers to a larger family of organic compounds synthesized by plants with structural phenolic features ranging from simpler phenolic acids to complex structures such as proanthocyanidins (Tijjani et al., 2020). Among the different classes of polyphenols, flavonoids and phenolic acids were experimentally investigated to show resistance against CT toxicity both through *in vitro* and *in vivo* studies (Saito et al., 2002; Cherubin et al., 2016).

*Careya arborea* (Rahman et al., 2003)*, Punica granatum* (Zhao et al., 2018), *Psidium guajava* (Birdi et al., 2010) are traditionally used in treating diarrheal infections in India and have also been scientifically validated to control and manage diarrheal infections caused by enteric pathogens such as *Shigella flexineri*, *Escherichia coli*, and *V*. *cholerae*. All the three selected plants showed effective neutralization against CT toxicity *in vitro* and *in vivo* from our earlier reported studies (Charla et al., 2022). Hydro-alcoholic extracts of these plants significantly reduced CT-induced elevated cellular cAMP levels in CHO cell line and also fluid accumulation in ligated-ileal loops of adult mouse. As the initial and critical step in CT pathogenesis involves its binding to the GM1 receptor on intestinal epithelial cells for internalization, we proposed to investigate and derive the role of selected phenolic acids and flavonoids from these plants in neutralizing the CT-induced toxicities by using both molecular modelling and experimental studies.

## 2 Methods

### 2.1 *In silico* studies

#### 2.1.1 Mining of bio-active compounds and molecular docking

From the three selected plants, polyphenolic compounds were mined from public database resources like PCIDB (Phytochemical interactions DB), Dr. Dukes DB, and literature search. Structural and chemical information of these phytocompounds were obtained from PubChem small molecule database (https://pubchem.ncbi.nlm.nih.gov/). This compiled dataset of bioactive compounds was filtered for duplicates and ubiquitous molecules.

**Figure 1:**
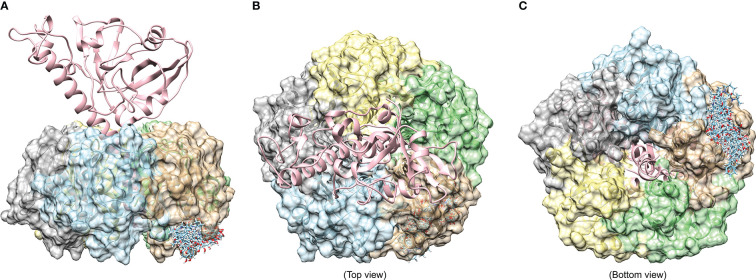
Binding of 20 phytocompounds to the largest binding pocket at chain F of 1XTC. **(A)** shows the front view, while the top **(B)** and bottom view **(C)** are also shown to highlight the orientation and architecture of whole CT having one alpha and five subunits of beta chain.

Virtual screening was performed using Dock6.9 software developed by UCSF (Allen et al., 2015). We used a flexible docking approach for virtual screening as most of the phytocompounds had larger molecular weight and would help to accurately predict the binding mode as well as intermolecular interactions of selected phytocompounds with CT. The three dimensional structure of CT was retrieved from the RCSB structural database (PDB ID: 1XTC) that has five β-subunits bound to the one α-subunit. We prepared the CT structure by adding missing residues and refining the generated model by performing energy minimization using steepest descent algorithm followed by conjugate gradient. The grid was set at the region where CT was reported to bind with GM1 receptor and the binding pocket residues were selected from the earlier reported structures (Merritt et al., 1994). Amongst all the five chains of β-subunits, we chose chain F (shown in brown surface representation in **Figure 1**) which showed a larger cavity at the GM1 binding interface and hence considered it as a potential binding site.

The default parameters for flexible docking were selected in this study. Selected 20 compounds were prepared for docking by adding hydrogen and converting them into ‘mol2’ file format using Openbabel2.4. The ligand conformations were ranked by considering their grid score. Best docked conformations were selected by analysing their intermolecular interactions with the active site of CT causing the disruption of CT binding to the GM1 receptor.

#### 2.1.2 Drug-likeness prediction

Drug likeness prediction and side effects of top 10 hits with 1XTC were performed by submitting the canonical SMILES of each molecule to an online prediction server ADMETlab 2.0 (Xiong et al., 2021).

#### 2.1.3 Molecular dynamics simulation of prioritized docked complexes

Based on the drug-likeness character, least grid score, and intermolecular interactions with active site residues, we prioritized two phenolic acids; Chlorogenic acid (*C. arborea, P. granatum*, and *P. guajava*) and Ellagic acid (*C. arborea*, *P. granatum* and *P. guajava*) and two flavonoids; Rutin (*P. granatum* and *P. guajava)*, Phloridzin (*P. guajava* and *P. granatum*) for further MD simulation studies along with their respective standards Gallic acid and Quercetrin. The stability of these six complexes were studied by performing all-atom MD simulation in explicit solvent using gromacs *ver.* 2021 (Abraham et al., 2015; Khanal et al., 2022; Khanal et al., 2023). The partial charges on the ligands were generated using antechamber module of AmberTools21 and x-leap was used to generate a topology file for the entire complexes with Amber-99SBildn forcefield.

Complexes were solvated using TIP3P water model and systems were neutralized by adding the required number of counter ions. These solvated systems were subjected to energy minimization using the steepest descent followed by a conjugate gradient algorithm. These energy minimized systems were equilibrated using canonical ‘NVT’ and isothermal-isobaric ‘NPT’ ensembles where temperature and pressure kept constant to 300K and 1 bar, respectively. Further, unrestrained all-atom explicit MD simulations were performed for 100ns, and trajectories at every 2fs were recorded. The parameters for MD simulations were taken from earlier similar studies (Bhandare and Ramaswamy, 2018; Gangopadhyay et al., 2019; Dwivedi et al., 2022; Patil et al., 2022). The trajectories obtained were analysed using inbuilt gromacs tools, chimera was used to analyse individual snapshots, and generate publication quality images. The tool *‘g_mmpbsa’* was used to calculate the relative binding energy using MM/PBSA approach (Kumari and Kumar, 2014). Intermolecular interactions were investigated using Biovia Discovery studio visualizer 2021.

### 2.2 *In vitro* studies

#### 2.2.1 Quantification of selected polyphenols

Bark of *C. arborea*, young leaves of *P. guajava* and fruit peel of *P. granatum* were collected in the Belagavi district of Karnataka, India. The collected plants and their parts were identified and authenticated by a certified plant taxonomist at ICMR-NITM, Belagavi. The voucher specimens were deposited in ICMR-NITM under accession numbers 1590 (*C. arborea*), 1690 (*P. granatum*), 1589 (*P. guajava*). Extraction of these powdered plant parts was carried out using 70% ethanol (v/v) in water by cold maceration technique (Ćujić et al., 2016). Six herbal compounds, including three phenolic acids, Ellagic acid (EA), Chlorogenic acid (CHL), Gallic acid (GA), and three flavonoids; Rutin (RTN), Phloridzin (PHD), and Quercetrin (QTRN), selected using *in silico* studies were procured from Sigma Aldrich. Quantification of these six compounds in hydro-alcoholic extract was performed using High-Performance Thin-Layer Chromatography (HPTLC).

#### 2.2.2 HPTLC analysis

CAMAG-HPTLC system was used for detection, separation and densitometric analysis of hydro-alcoholic extracts of *C. arborea*, *P. guajava*, *P. granatum* for selected six compounds (Pai et al., 2014; Pai and Joshi, 2018). The stock solutions of extracts and standards were prepared as 1.0 mg/mL in methanol except EA which was dissolved in 99.7% DMSO. Analysis was performed on a pre-coated TLC silica gel G60 F_254_ plates (ALUGRAM) (10 × 10 cm) for phenolics (EA, CHL and GA) and RP-18W/UV_254_ (ALUGRAM) (10 × 10 cm) for flavonoids (RTN, PHD and QRTN). Standards and sample bands (6 mm) were applied using CAMAG Linomat-5 TLC Sampler using 100 μl syringe. Band length 6 mm, application rate 50 μL/s distance from the bottom of the plate (Y) 10 mm, distance from edge of plate (X) 10 mm, distance between bands were auto set. The plates were developed up to a distance of 80 mm with toluene: ethyl acetate: methanol: formic acid in the ratio of 3:3:0.8:0.6 v/v (for phenolics) and tetrahydrofuran: water: formic acid in the ratio of 4: 6: 0.5 v/v (for flavonoids) as mobile phase. The details of peaks were recorded at 280 nm (for phenolics) and 310 nm (for flavonoids) using CAMAG TLC scanner 3 and WinCATS–Planar Chromatography Manager, (Version 1.4.4.6337), Switzerland.

#### 2.2.3 Estimation of CT concentration

Cell free culture filtrate (CFCF) of clinical strains of *V. cholerae* collected during cholera outbreaks in the years 2010 and 2012 from the southern Indian state of Karnataka, India were used in the current study (Roy et al., 2012; Dey et al., 2014). A single isolate efficiently secreting CT into CFCF was screened and selected using GM1 ELISA. CT procured from Sigma Aldrich was used as the standard to plot the CT standard curve. The clinical isolate of *V. cholerae* was cultured in AK1 media to stimulate the production of CT as described (Iwanaga et al., 1986). Initially, MH broth was inoculated with a pure culture of *V. cholerae* and grown for 12 hrs at 37°C. Then the inoculum size in MH broth was adjusted to 10^5^-10^7^cfu/mL, 1mL of this inoculum was added to 10mL of AK1 media in stationary tubes followed by incubation for 4hrs and subsequently transferred to a flask kept on a shaker at 150rpm for 16hrs at 37°C. CFCF was obtained by centrifuging the culture at 12000 rpm for 20 min and filtering the obtained culture supernatant through a sterile 0.22μm syringe filter. This unconcentrated CFCF was stored as multiple aliquots at -20°C until further use to prevent repeated freeze-thawing as it affects CT concentration in CFCF.

Pure CT reconstituted as 5mg/mL in deionized water was taken at known concentration from 1250ng/mL and diluted two-folds upto 39ng/mL whereas CFCF was taken at different dilutions (1:5; 1:10; 1:20 and 1:30) in PBS (pH7.4) and both were added to 96 micro-titer well pre-coated with GM1 (1.44μM). Distilled water and AK1 media were used as solvent controls for CT and CFCF respectively and the total volume in each well was made to 100μL with PBS. Initial incubation was carried out for 1 hr at 37°C, and GM1 ELISA was performed as described (Svennerholm and Wiklund, 1983) using polyclonal rabbit anti-CT antibody diluted 1:600 and anti-rabbit IgG-peroxidase diluted 1:200 in PBS procured from Sigma Aldrich. Absorbance was recorded at 492nm and the concentration of CT in CFCF was determined from the standard curve. This CFCF with quantified CT concentration was used for all the *in vitro* and *in vivo* experiments.

#### 2.2.4 MTT cytotoxicity assay

CHO cell line used for analysing cytotoxicity of six herbal compounds was procured from NCCS, Pune. Cytotoxicity of the six compounds on the CHO cell line was determined using the MTT assay as described (Plumb et al., 1989). CHO cells were seeded at a density of 20,000 cells/well and incubated for 24 hours at 37°C and 5% CO_2_. The stock solutions of CHL, GA, RTN, PHD, and QRTN were prepared as 2 mg/mL in 0.05% DMSO, whereas EA stock was prepared as 2 mg/mL in 99.7% DMSO and filtered using 0.22μm syringe filters. After 24hrs, CHO cells were treated with increasing concentrations of six compounds, ranging from 18 to 700μg/mL each, in triplicates in DMEM media supplemented with 2% FBS and incubated for 24hrs at 37°C and 5% CO_2_. Simultaneously, untreated cells and cells treated with DMSO were taken as cell control and vehicle control, respectively. Subsequently, cells were washed twice with PBS and treated with 20μL of 0.5% MTT solution in PBS for 4 hours, and formazan crystals formed after 4 hours were solubilized using DMSO. The absorbance was taken at 570nm and the IC_50_ of six compounds on the CHO cell line was estimated using the cytotoxicity percentage.

Using IC_50_ values, two non-cytotoxic concentrations (NC1 and NC2) for each of the six compounds were determined by using 25% and 12.5% of their respective IC_50_ values, and the viability percentage was estimated by the MTT assay.

#### 2.2.5 Binding inhibition percentage

The binding inhibition of CT to GM1 by selected phenolic acids (EA and CHL) and flavonoids (RTN and PHD) along with their respective standards was determined by GM1 ELISA as described earlier. Initially, the CT concentration in CFCF was estimated from the CT standard curve. CFCF titer with a CT concentration of 3200ng/mL was diluted twofold up to 100ng/mL to plot the CFCF absorbance curve and to select the CT concentration exhibiting maximum absorbance at the highest dilution. The selected CFCF titer was treated with NC1 and NC2 of each of the six compounds and incubated on a shaker at 120rpm for four hours at 37°C. Later, this pre-incubated mixture was added along with CFCF control to a pre-coated GM1 96-well micro-titer plate, and GM1 ELISA was performed in triplicates. Simultaneously, each concentration of six compounds was also added in triplicates to get the background measurements, and the binding inhibition percentage was calculated.


$$BindingihibitionBI\%=1-\frac{A_{test}}{A_{cfcfcontrol}}\times 100$$

#### 2.2.6 Cyclic AMP assay

Elevated cAMP levels in CT-induced CHO cell lines were evaluated using the abcam cAMP *in vitro* competitive ELISA kit as per the manufacturer’s instructions. CHO cells seeded at a density of 4x10^4^ cells/well in a 96-well microtiter plate were treated with a pre-incubated mixture containing CFCF (CT=100ng/mL) and each of six compounds at NC1 and NC2 in duplicates. Untreated CHO cells and cells treated with CFCF alone were taken as cell control and positive control, respectively. Concurrently, cells were also treated with each of the six compounds to establish that the selected compounds have no effect on the intracellular cAMP levels. The inhibitory activity of six compounds against CT-induced cAMP levels in CHO cell line was evaluated as an increase in bound percent (B%) of cAMP *w.r.t.* CFCF control.

#### 2.2.7 Protective activity against CT-induced cell elongation

The protective activity of six compounds against CT-induced cell elongation in CHO cell line was analyzed as described by Saha et al., 2013. CHO cells were seeded at a density of 6x10^4^/well in 12-well culture plates supplemented with 10% FBS + DMEM media and incubated for 24hrs at 5% CO_2_ and 37°C. CFCF titer with CT concentration 100ng/mL was treated with NC1 and NC2 of each of six compounds and incubated at 37°C under shaking conditions at 120rpm for 4hrs. After 24 hrs, the pre-incubated mixture was added to CHO cells in duplicates supplemented with 2% FBS+DMEM media and further incubated for 18 hrs at 5% CO_2_ and 37°C. CHO cells were also simultaneously treated with six compounds alone to check their effect on cell morphology. After 18 hrs, cells were washed twice with PBS, replenished with fresh media, and observed for morphological changes under a phase contrast microscope.

### 2.3 *In vivo* studies

Based on *in vitro* results, two phenolic acids EA and CHL along with standard GA were further investigated by *in vivo* studies.

#### 2.3.1 Mice ligated-ileal loop assay

The ligated-ileal loop assay using adult Swiss albino mice was performed as described by Sawasvirojwong et al., 2013 to investigate the neutralizing activity of three phenolic acids, i.e. EA, CHL, and GA against CFCF-induced fluid accumulation. This animal study was approved by the Institutional Animal Ethical Committee of ICMR-NITM, Belagavi (Accession number: IAEC/ICMR-NITM BGM/2018/07). Six-week old Swiss albino mice weighing 25-30g were procured from the laboratory animal facility of MMDC, Belagavi, Karnataka and acclimatized under controlled conditions of temperature, humidity, and light at the Animal Research Facility of ICMR-NITM, Belagavi.

A total of 48 Swiss albino mice were randomized into 8 groups (n =6) and the grouping is detailed as (A) Saline control (Untreated): received 100ul of PBS/loop; (B) CFCF control: received CFCF with CT concentration (1µg/loop); (C) EA1: 50µg of EA + CFCF (CT 1µg)/loop; (D) EA2: 25µg of EA + CFCF (CT 1µg)/loop; (E) CHL1: 50µg of PGRPE + CFCF (CT 1µg)/loop; (F) CHL2: 25µg of CHL + CFCF(CT 1µg)/loop; (G) GA1: 50µg of GA + CFCF(CT 1µg)/loop; (H) GA2: 25µg of GA + CFCF (CT 1µg)/loop. Group C-H received a pre-incubated mixture of each of three herbal compounds at two variable concentrations with CFCF in each ligated-ileal loop.

Prior to the experiment, mice were fasted overnight, given free access to water, and later anaesthetized by Intraperitoneal injection of Ketamine-Xylazine (87.5mg/kg; 12.5mg/kg, respectively). A loop of distal ileum approximately 2 cm in length was exteriorized by making a small abdominal incision and tying at both ends using surgical thread. Without causing any injury to the tissue, these ligated-ileal loops were injected with a PBS/CFCF/pre-incubated mixture of three compounds with CFCF, gently restituted, and sutured. After 18 hrs, mice were sacrificed and ileal loops were excised. The protection of EA, CHL, and GA against CFCF-induced fluid accumulation was evaluated as the mean weight/length ratio of ligated-ileal loops.

#### 2.3.2 Estimation of cAMP in ligated-ileal loops

The inhibitory activity of three phenolic acids against CFCF-induced cAMP levels in ligated-ileal loops was estimated using abcam competitive ELISA as per the manufacturer’s instructions. After 18 hours, the weight of ileal tissues excised from sacrificed mice was measured and stored at -80°C. Prior to the experiment, the ileal tissues from each group (A to H) were pooled and 100mg of tissue was homogenized using lysis buffer. The supernatant collected by centrifuging the homogenized tissue at 12000rpm for 10 min at 4°C was assayed for cellular cAMP levels.

#### 2.3.3 Histopathology

Histological analysis of mice ligated-ileal loops was carried out by initially fixing them in 10% formalin and then the intestinal tissue sections were stained using hematoxylin and eosin. The histopathological changes were investigated as described by Sawasvirojwong et al. (2013) at 40X magnification using light microscopy.

### 2.4 Statistical analysis

The Grace 5.1.25 software package was used to represent all the plots generated by analysing the MD trajectories. The results from *in vitro* experiments were expressed as mean ± standard deviation (n = 3) and those of *in vivo* experiments as mean ± standard error (n = 6). The difference in means between the control and test groups was analyzed using One-Way ANOVA followed by the Dunnett test (Graphpad Prism *ver.* 5.0), and *p<0.05 is considered statistically significant.

## 3 Results

### 3.1 *In silico* studies

#### 3.1.1 Mining of bio-active compounds and molecular docking

Previously reported 20 polyphenolic compounds were chosen from three plants, excluding the pervasive molecules (**Supplementary Table 1**). The docking study reveals the stable binding of selected 20 compounds to the preferred binding site (expected to bind to the GM1 receptor) with varying binding affinity. The grid score cut off for flavonoids and phenolic acids was selected based on grid scores of standards QRTN (-37.249) and GA (-28.355) (**Supplementary Tables 2 and 3**). We have carefully analyzed the top 10 hits (including standard-gallic acid) to get more structural insights into their intermolecular interactions. All the selected 10 top hits from the flexible virtual screening show maximum binding affinity when compared to the standard GA. **Table 1** represents the list of top 10 hits, their grid scores, and intermolecular interactions. The maximum number of combined H-bonding and other interactions are observed in the complex CT-Ellagic acid (10), followed by CT-Procyanidin B3 (9) and CT-Delphinidin (9).

**Table 1:** List of top 10 hits interacting with active site residues of 1XTC (chain F) binding to GM1 receptor using Dock6.9.

Compound name	Grid Score	Hydrogen bond interactions (No. of interactions)	Van der Waals, Pi-Alkyl, CH, Pi-Cation, Pi-Sigma, Pi-Pi stacked, Pi-Pi T-shaped interactions (No. of interactions)	Active site residues within interactions	Total no. Of interactions with active site residues
Rutin (Flavonoid)	-48.042	Asn14, Gln61, Glu51, Gln56(2)	His13	Asn14, GLN61, Glu51, Gln56, His13	6
Phlorizin (Flavonoid)	-44.3875	Asn90, Asn14, Glu51	His57, Trp88(2), Ile58	Asn90, Asn14, Glu51 Trp88, Ile58	6
Phellatin (Flavonoid)	-43.699	Glu51, Glu11, Glu56, Gln61	Trp88, His13(2)	Glu51, Glu11, Glu56, Gln61 Trp88, His13	7
Procyanidin B2 (Flavonoid)	-43.4793	His13, Gln61, Glu51, His57, Gln56	Trp88(3), Ile58	His13, Gln61, Glu51, Gln56, Trp88(3), Ile58	8
Procyanidin B1 (Flavonoid)	-42.4149	Trp88, Glu51(2), Ser55, Asn90	Trp(88)	Trp88, Glu51, Asn90	5
Procyanidin B3 (Flavonoid)	-40.0482	Gly33, Gln61, Glu51, Gln56, Glu11(2), His57	Trp88(2), Ile58	Gly33, Gln61, Glu51, Gln56, Glu11, Trp88, Ile58	9
Chlorogenic acid (Phenolic acid)	-39.2645	Gly33, Gln61, Trp88, Lys91, Gln56(3), Gln61	Nil	Gly33, Gln61, Trp88, Lys91, Gln56, Gln61	8
Delphinidin (Flavonoid)	-38.1791	His13(2), Asn90, Glu51, Gly33	Trp88(4)	His13, Asn90, Glu51, Gly33, Trp88	9
Quercetrin (Flavonoid)	-37.2493	Asn14, Gln61, Gln56	His57, Trp88	Gln61, Gln56, Trp88	3
Ellagic acid (Phenolic acid)	-36.4194	Gln61(2), Asn90(2), Glu51, Gly33, His57	Trp88(5)	Gln61, Asn90, Glu51, Gly33, Trp88	10
Gallic acid (Phenolic acid)	-28.355	Gln61(2), Asn90(2), Glu51, His57	Trp88(2)	Gln61, Asn90, Glu51, Trp88	7

#### 3.1.2 Drug-likeness and side effects

**Figure 2:**
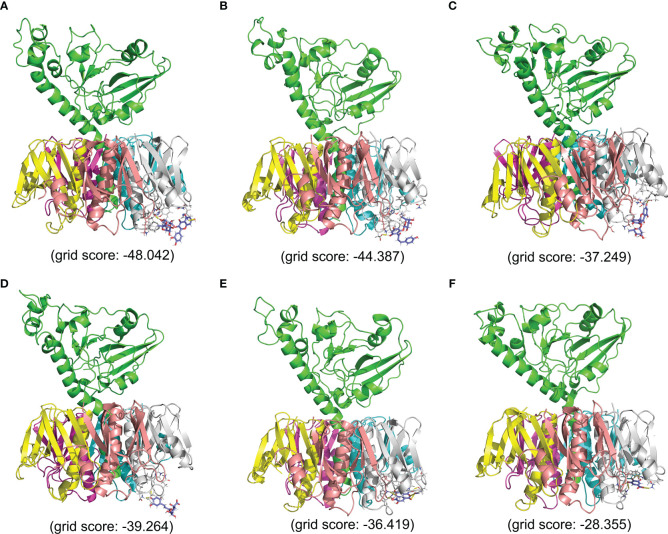
The 3D representation of binding mode and the interactions of the six selected compounds with 1XTC with their respective grid score. **(A)** CT-RTN, **(B)** CT-PHD, **(C)** CT-QRTN, **(D)** CT-CHL, **(E)** CT-EA, **(F)** CT-GA.

**Figure 3:**
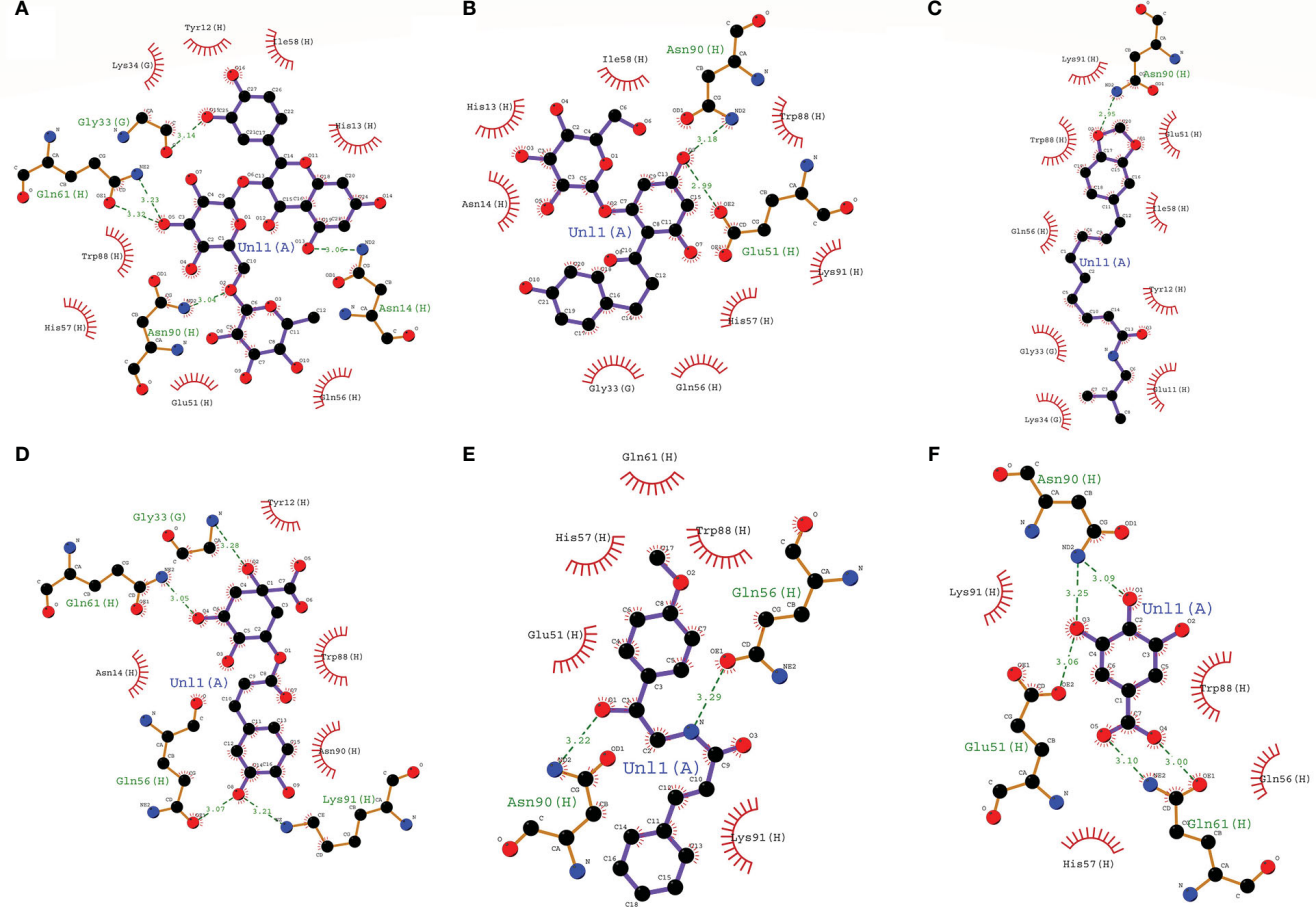
The 2D representation of the binding mode and atomic level intermolecular interactions of six selected compounds with 1XTC. **(A)** CT-RTN, **(B)** CT-PHD, **(C)** CT-QRTN), **(D)** CT-CHL, **(E)** CT-EA, **(F)** CT-GA.

The ADMET profile and drug-likeness of the top 10 hits are listed in **Supplementary Table 4**. Phenolic acids showed a more favorable ADMET profile and drug-likeness compared to the flavonoids. From among the top 10 hits, we selected two phenolic acids and two flavonoids complexes with their respective standards based on their grid score and drug-likeness for further molecular dynamics simulation studies (CT-Ellagic acid, CT-Chlorogenic acid, CT-Gallic acid, CT-Rutin, CT-Phloridzin, and CT-Quercetrin). These complexes are abbreviated as CT-EA, CT-CHL, CT-GA, CT-RTN, CT-PHD, CT-QRTN, and these abbreviations are used in the entire manuscript hereafter. The binding modes (in 3D) and their atomic level intermolecular interactions (2D) for these six selected complexes are represented in **Figures 2**, **3**.

#### 3.1.3 Structural stability of the docked complexes using MD

**Figure 4:**
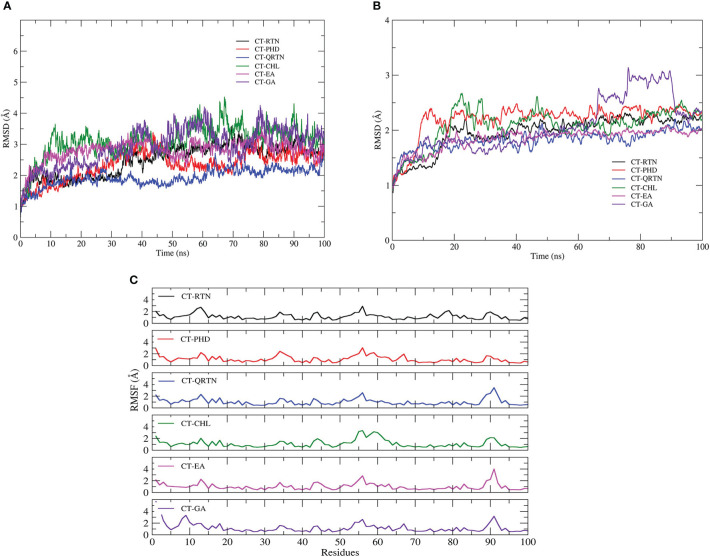
**(A)** The structural stability of the simulated complexes analyzed by plotting backbone RMSD of CT-complexes **(B)** RMSD of chain F i.e. GM1 binding pocket **(C)** Residual fluctuations of GM1 binding pocket.

**Figure 5:**
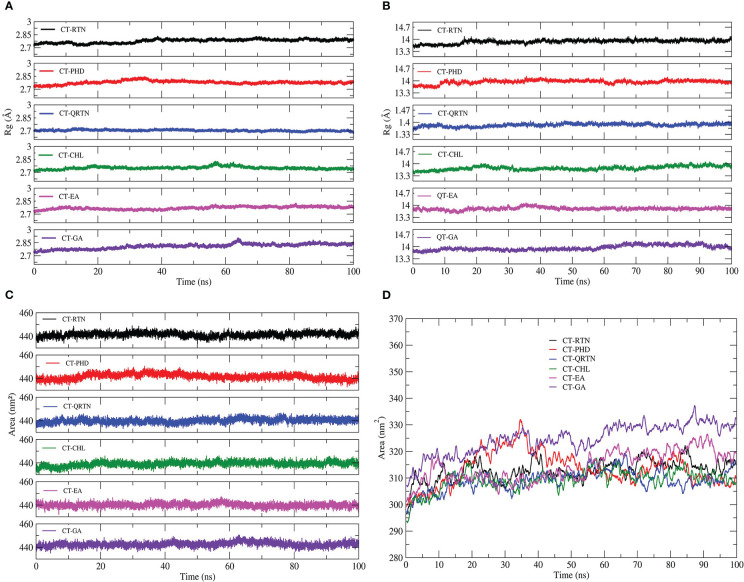
The compactness of the CT-complexes and chain F GM1 binding pocket is shown as **(A)** and **(B)** respectively. The hydrophobicity of CT-complexes is also quantified by analysing solvent accessible surface area for the entire CT-complex **(C)** as well as chain F **(D)**.

Molecular dynamics simulations are used to investigate the structural stability and intermolecular interactions of the six complexes: CT-EA, CT-CHL, CT-GA, CT-RTN, CT-PHD, and CT-QRTN. The quality check was performed over the 100 ns trajectory generated using gromacs by analyzing potential energy of the simulated systems. The equilibration of the simulated complexes was confirmed by plotting the temperature and pressure over the MD simulation trajectory. It is observed that the temperature, pressure, and potential energy of the simulated complexes are stable with no considerable fluctuations. The temperature and pressure are maintained constant at 300K and 1bar, respectively, during the simulation in all the studied complexes (**Supplementary Figure 1**). The average backbone RMSD value of these six complexes ranges between 1.5 and 3.5Å. It was observed that these complexes are well equilibrated during the 0 to 60 ns of the simulation and thereafter show stable dynamics throughout the simulation period (**Figure 4A**). The RMSD of chain F is stabilized at a value below 2.25Å except for the CT-GA complex, which showed a maximum RMSD value after 60 ns of simulation time (**Figure 4B**). The complex CT-EA and CT-PHD show the lowest RMSF values except for the flexible loop at the binding pocket region in the CT. The residues participating in the non-bonded interactions showed fewer fluctuations as their flexibility was arrested by stable intermolecular interactions (**Figure 4C**). The R*g* value, analyzed for the entire CT complex and separately for Chain F, also revealed the stable dynamics during the 100ns simulation in all the complexes (**Figures 5A, B**). A similar observation was made for solvent accessible surface area analysis of the overall CT complex. However, variations in the SASA value of chain F (GM1 binding pocket) were observed during the simulation. The graphical representation of SASA with for CT and chain F is shown in **Figures 5C, D**, respectively.

#### 3.1.4 Intermolecular interactions in docked complexes

**Figure 6:**
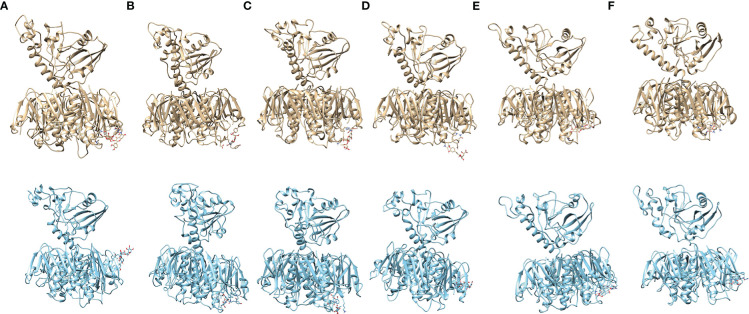
The MD simulation snapshots revealing the stable complex formation during the 100ns of simulation. The starting MD conformations are shown in gold (A1-F1) while MD simulation end structure (100ns) are shown in cyan (A2-F2), where **(A–F)** represents complexes CT-RTN, CT-PHD, CT-QRTN, CT-CHL, CT-EA and CT-GA respectively.

**Figure 7:**
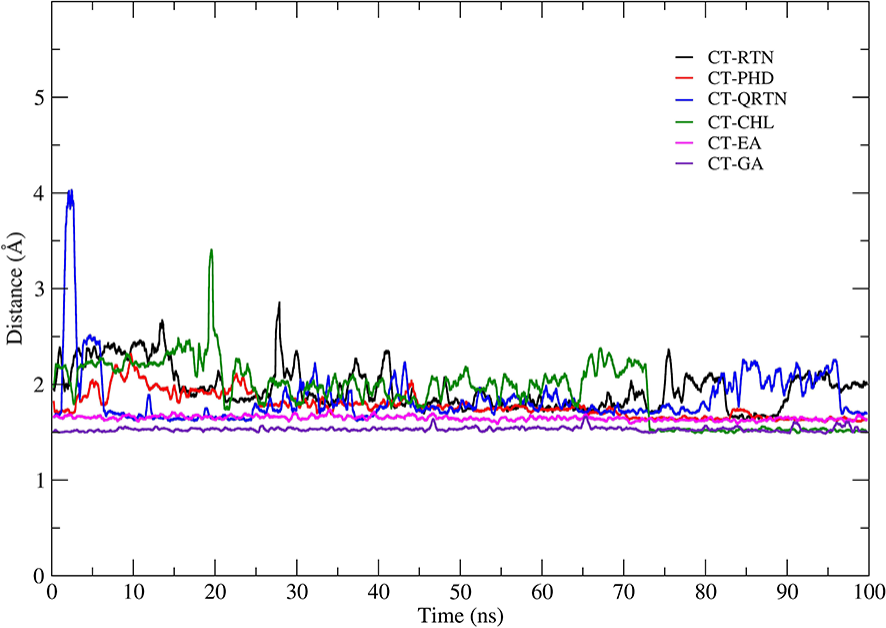
The variations in the minimum distance observed between the GM1 binding pocket (chain F) with the six phytochemicals during the simulation.

From structural stability analysis, it is observed that docked complexes are well stabilized during the simulation. Therefore, we characterized intermolecular interactions in the initial and final MD simulation conformations. Furthermore, we also noted the intermolecular interactions precisely with the active site residues. The maximum number of H-bonds formed between CT and ligands during the MD simulations in all the six complexes is shown in **Supplementary Figure 2**. The list of all the non-bonded interactions (H-bonds, hydrophobic interactions, etc.) that trigger stable complex formation before and after simulations is given in **Supplementary Table 5**. Total non-bonded interactions are increased in the complexes CT-PHD (6 to 9) and CT-QTRN (3 to 12), whereas other complexes showed a decrease in the total non-bonded interactions in initial and final MD conformations. This revealed the consistent interactions with the binding pocket residues are increased in the complexes CT-PHD and CT-QRTN, whereas in other complexes these interactions are not consistent. The initial (0 ns) and final (100 ns) snapshots of the MD simulation trajectory are shown in **Figure 6**. To gain more structural insights into these intermolecular interactions, we plotted the minimum distance between the six compounds and CT chain F (**Figure 7**). It showed the distance of CT-EA and CT-PHD is constant <2.5Å, whereas in other complexes it showed significant fluctuations (up to 7Å). The relative binding energy of six complexes was calculated using the MM/PBSA approach and is represented in **Table 2**. The order of binding free energy was observed as CT-EA > CT-CHL > CT-GA for phenolic acids and CT-PHD > CT-QRTN > CT-RTN for flavonoids. The selected polyphenolic compounds showed much higher binding affinity than their respective controls. This highlights the potency of these compounds in the successful inhibition of CT.

**Table 2:** Calculation of binding free energy (in kcal/mol) of six complexes using *‘gmx_MMPBSA’* tool.

Complexes	ΔE_VDW_	ΔE_ELE_	ΔE_GB_	ΔE_SURF_	ΔG_GAS_	ΔG_SOLV_	ΔTOTAL
CT-EA	-31.86 ± 0.02	-34.04 ± 2.61	42.68 ± 0.58	-3.78 ± 0.01	-65.9 ± 2.61	38.9 ± 0.58	-27 ± 2.67
CT-CHL	-7.86 ± 0.28	-78.25 ± 4.2	62.39 ± 1.69	-2.11 ± 0.02	-86.11 ± 4.21	60.27 ± 1.69	-25.83 ± 4.54
CT-GA	-7.27 ± 0.01	-81.88 ± 1.57	67.01 ± 0.55	-2.1 ± 0	-89.15 ± 1.57	64.91 ± 0.55	-24.25 ± 1.66
CT-PHD	-33.33 ± 0.65	-76.09 ± 2.98	80.48 ± 3.16	-5.21 ± 0.06	-109.42 ± 3.05	75.27 ± 3.16	-34.16 ± 4.39
CT-QRTN	-28.5 ± 1.26	-38.14 ± 3.51	42.6 ± 1.33	-4.03 ± 0.03	-66.64 ± 3.73	38.57 ± 1.33	-28.07 ± 3.96
CT-RTN	-24.62 ± 1.18	-19.52 ± 8.84	29.71 ± 3.92	-3.31 ± 0.21	-44.14 ± 8.92	26.4 ± 3.92	-17.74 ± 9.75

ΔE_VDW_, van der Waals contribution; ΔE_ELE_, electrostatic energy; ΔE_GB_, electrostatic contribution to the solvation free energy; ΔG_SURF_, solvent-accessible surface area; ΔG_SOLV_, polar solvation free energy; ΔTOTAL, Binding free energy.

#### 3.1.5 Secondary structural changes during simulation

We analyzed conformational changes at the binding site over the simulation period using the DSSP tool. **Supplementary Figure 3** represents the distortions in the secondary structure calculated in all the complexes (A-F). The binding pocket architecture was well stabilized in complexes CT-EA and CT-PHD compared to other complexes, mainly due to the increased helical content in the chain-F (also refer to **Supplementary Videos 1-6**).

#### 3.1.6 Contribution of the binding pocket residues in the intermolecular interactions

The residue decomposition energy calculated over the stable trajectory revealed the contribution of individual resides in the binding energy (**Supplementary Figure 4**). The positive contribution energy refers to the specific residue that does not favor the interactions, while the negative contribution energy refers to favorable interactions, binding pocket residues show the least binding energy. Most of the residues in complexes CT-EA, CT-PHD, and CT-CHL showed negative contribution energy. But in other studied complexes, the contribution energies varied a lot, which makes it hard for them to form stable complexes.

### 3.2 *In vitro* studies

#### 3.2.1 Quantification of selected polyphenols using HPTLC

Quantitative analysis of phenolics (EA, CHL and GA) and flavonoids (RTN, PHD and QRTN) in in hydro-alcoholic extracts of *C. arborea*, *P. guajava* and *P. granatum* performed using HPTLC is represented in **Table 3**. The HPTLC-PDA chromatogram are represented separately for phenolic acids and flavonoids in **Supplementary Figure 5**.

**Table 3:** HPTLC quantitative analysis of phenolics (EA, CHL and GA) and flavonoids (RTN, PHD and QRTN) in (μg/mg).

Compound name	*C. arborea*	*P. guajava*	*P. granatum*	Linearty
Ellagic acid	0.3 ± 0.01	0.7 ± 0.02	10.30 ± 0.76	y = 47780x - 1459.5
Chlorogenic acid	0.4 ± 0.05	0.45 ± 0.03	0.6 ± 0.02	y = 14317x - 37.442
Gallic acid	458.25 ± 26	732.4 ± 19.7	ND	y = 6610.2x + 1578.99
Rutin	ND	2.31 ± 0.15	19.70 ± 0.06	y = 7053.3x + 163.01
Phloridzin	ND	4.83 ± 1.06	ND	y = 6964.6x + 259.35
Quercetrin	ND	5.60 ± 1.5	20.60 ± 2.8	y = 8577.3x + 224.48

ND (Not detected).

#### 3.2.2 MTT cytotoxicity assay

**Figure 8:**
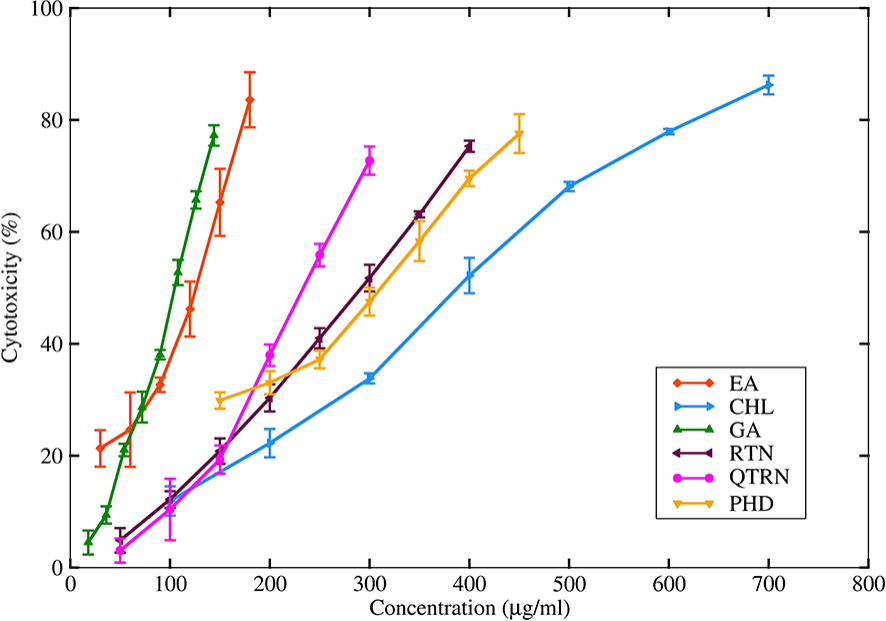
IC_50_ of six herbal compounds determined by MTT assay on CHO cell line.

Among the six selected compounds, GA was observed to be more cytotoxic to CHO cells with an IC_50_ of 102.7 ± 1.14μg/mL whereas CHL, with its IC_50_ value of 398.2 ± 5.4μg/mL showed the least cytotoxicity. The MTT assay revealed that two non-cytotoxic concentrations (NC1 and NC2) of each of the six compounds showed > 90% viability on the CHO cell line (**Figure 8**). **Table 4** lists the IC_50_ of six compounds along with their respective NC1 and NC2.

**Table 4:** The IC_50_ of six herbal compounds and their non-cytotoxic concentrations (NC1 and NC2).

Plant name	IC_50_ (μg/mL)	NC1(μg/mL)	Viability (%)	NC2 (μg/mL)	Viability (%)
Rutin (RTN)	287.4 ± 6.9	70	92.4	35	97.6
Phloridzin (PHD)	297.9 ± 3.9	75	94.8	37.5	97.6
Quercetrin (QRTN)	233.5 ± 3.7	60	91	30	96.3
Chlorogenicacid (CHL)	398.2 ± 5.4	100	90.5	50	97.2
Ellagic acid (EA)	115.5 ± 9.6	30	93.2	15	97.5
Gallic acid (GA)	102.7 ± 1.1	25	93.5	12.5	97.9

#### 3.2.3 Binding inhibition assay

**Figure 9:**
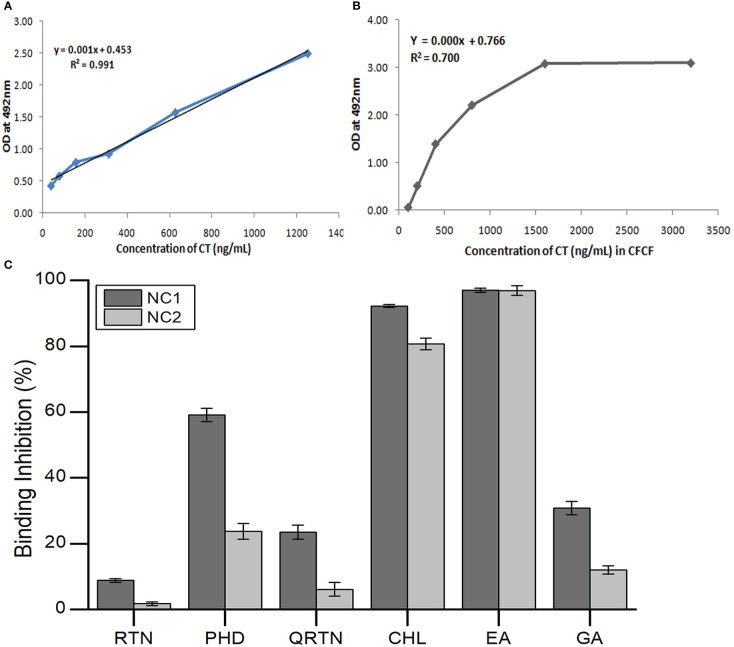
Binding inhibition percentage (BI%) of six herbal compounds to GM1. **(A)** CT calibration curve; **(B)** CFCF absorbance curve; **(C)** BI% of three phenolic acids (CHL, EA, GA) and three flavonoids (RTN, PHD, QRTN) to GM1 at their respective non-cytotoxic concentrations (NC1 and NC2) using GM1 ELISA.

Using the CT standard curve, the average concentration of CT in CFCF was calculated as 19.78 ± 0.52μg/mL. The maximum absorbance shown by the highest dilution of CFCF with a CT concentration of 1600ng/mL indicated the saturation of GM1 with CT. The selected six compounds (NC1 and NC2) were pre-incubated with CFCF titer with a CT concentration of 1600ng/mL and binding inhibition percentage (BI%) was evaluated using GM1 ELISA. Phenolic acids, EA, and CHL showed distinct activity against CT binding to GM1 compared to the standard GA, whereas, among flavonoids, PHD showed higher BI% (**Figure 9**).

#### 3.2.4 Cyclic AMP assay

**Figure 10:**
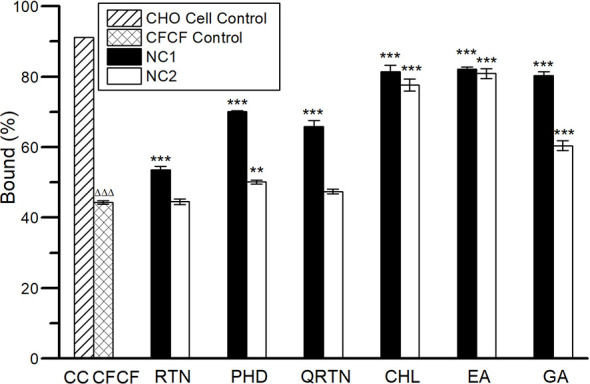
Efficacy of six herbal compounds against elevated cAMP levels in CHO cell line. *^ΔΔΔ^p*<0.001 compared with CHO cell control. **p<0.01 compared with CFCF control; ***p<0.001 compared with CFCF control.

The elevation of cAMP levels in the CHO cell lines was determined as the bound percent (B%) of cAMP using competitive ELISA (cellular cAMP levels are inversely proportional to B%) following kit instructions. Significant reduction in cAMP levels (***p<0.001) was observed for all three phenolic acids compared with CFCF control at both NC1 and NC2, whereas flavonoids showed a significant reduction in cellular cAMP levels (***p<0.001) at NC1 only (**Figure 10**).

#### 3.2.5 Protective activity against CT-induced cell elongation

**Figure 11:**
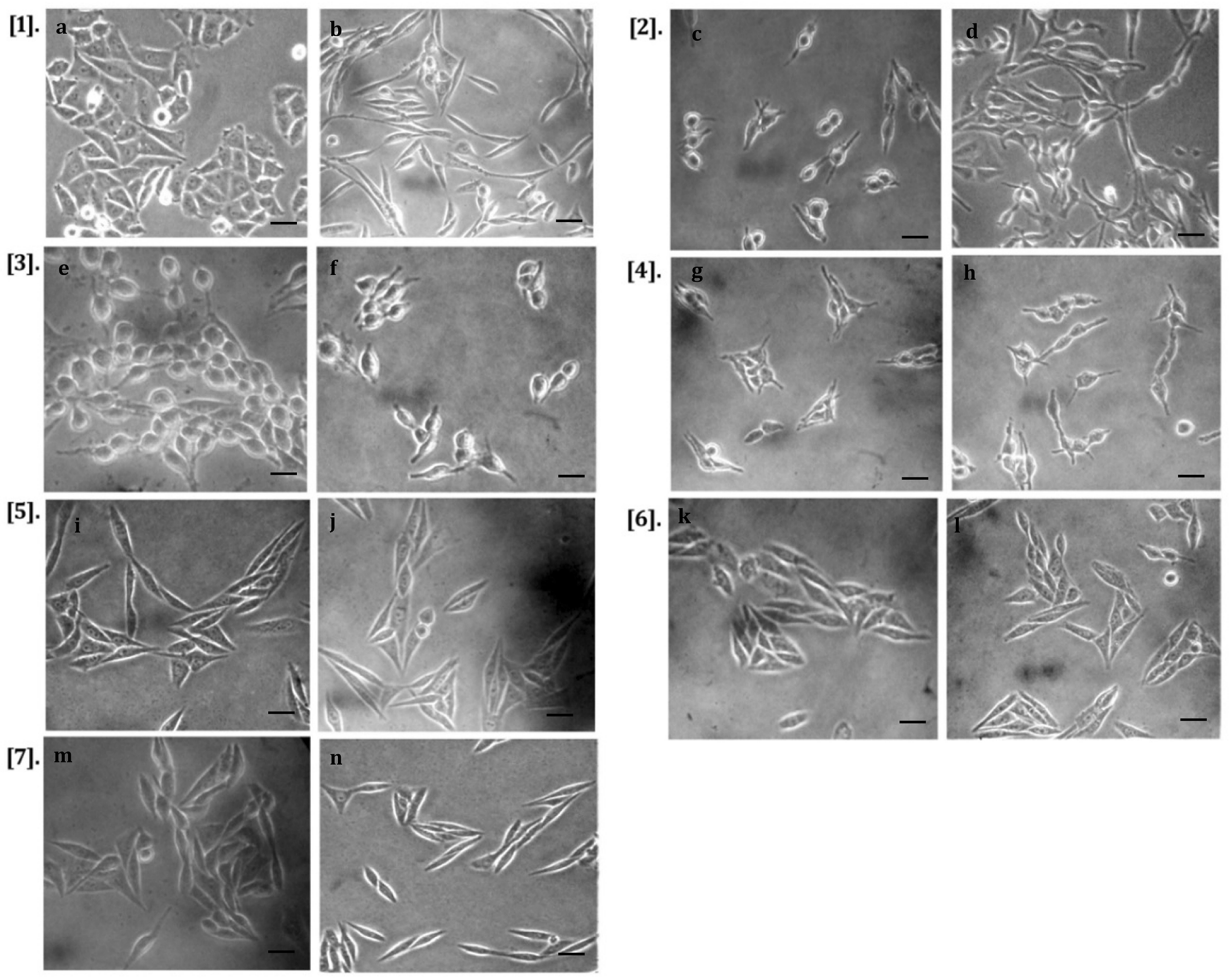
Protective activity against CT-induced cell elongation in CHO cell line. [1] **(A)** CHO cell control, **(B)** CFCF control (CT=100ng/mL); [2] **(C)** RTN (NC1)+CFCF, **(D)** RTN (NC2)+CFCF; [3] **(E)** PHD (NC1)+CFCF, **(F)** PHD (NC2)+CFCF; [4] **(G)** QRTN (NC1)+CFCF, **(H)** QRTN (NC2)+CFCF; [5] **(I)** EA (NC1)+CFCF, **(J)** EA (NC2)+CFCF; [6] **(K)** CHL (NC1)+CFCF, **(L)** CHL (NC2)+CFCF; [7] **(M)** GA (NC1)+CFCF, **(N)** GA (NC2)+CFCF at 40X magnification (scale bar 5µm).

The protective activity of phenolic acids and flavonoids against CFCF (CT=100ng/ml) induced cell elongation was investigated by phase contrast microscopy. All the selected compounds, excluding RTN, showed protection against CT-induced cell elongation at both NC1 and NC2. Further, EA and CHL showed greater protective activities among the selected six compounds (**Figure 11**).

### 3.3 *In vivo* studies

#### 3.3.1 Mice ligated-ileal loop assay

**Figure 12:**
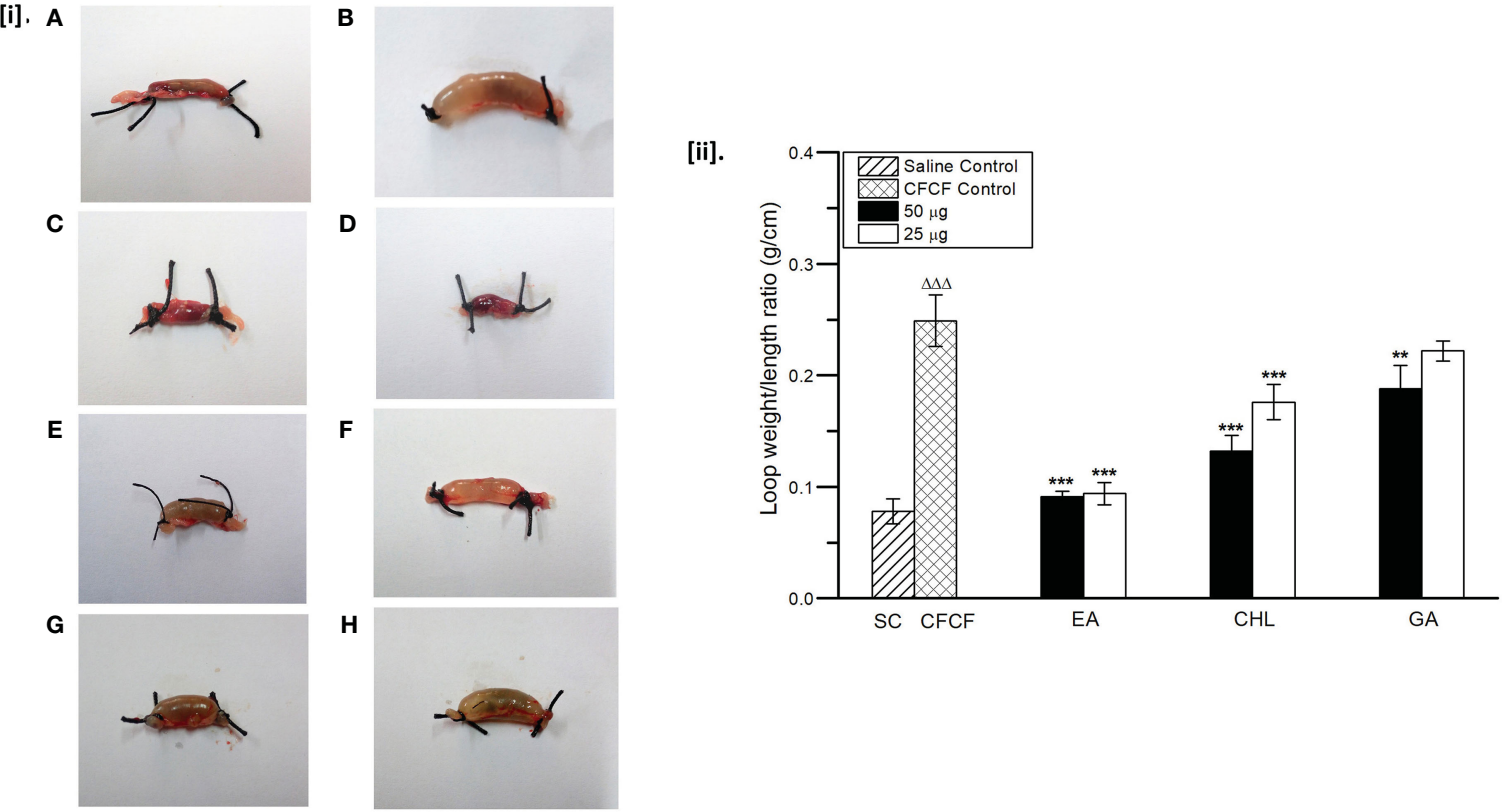
Protective activity of phenolic acids against CFCF induced fluid accumulation. [i]. **(A)** Saline control (100μl PBS), **(B)** CFCF control (CT=1μg/loop), **(C)** EA (50μg)+CFCF, **(D)** EA (25μg)+CFCF, **(E)** CHL(50μg)+CFCF, **(F)** CHL (25μg)+CFCF, **(G)** GA (50μg)+CFCF, **(H)** GA (25μg)+CFCF. [ii] Mean W/L ratio of mice ligated-ileal loops; All values were expressed as mean ± SEM (n=6), One Way Analysis of Variance (ANOVA) followed by Dunnett test. *^ΔΔΔ^p*<0.001 compared with saline control. **p < 0.01 compared with CFCF control; ***p < 0.001 compared with CFCF control.

Based on *in vitro* results, EA, CHL, and GA at two concentrations (50µg and 25µg/loop) were selected for further *in vivo* validation. The mean weight/length (W/L) ratio of saline instilled ileal loops was 0.078 g/cm ± 0.01 and that of CFCF treated ileal loops was calculated as 0.249 g/cm ± 0.02. Ligated-ileal loops of mice treated with CFCF containing 1µg of CT caused significant intestinal fluid accumulation after 18 hrs of the incubation period (*^ΔΔΔ^p*<0.001) compared to Saline control. Interestingly, both EA (0.091g/cm and 0.094g/cm) and CHL (0.132g/cm, 0.176g/cm) showed a significant decrease (***p<0.001) in mean W/L ratio at 50ug and 25µg respectively, whereas standard GA (0.188g/cm) showed a significant reduction (**p<0.01*)* at 50ug in fluid accumulation induced by CFCF compared to CFCF control (**Figure 12**). The mean W/L ratio of control and test groups is represented in **Supplementary Table 6**.

#### 3.3.2 Estimation of cAMP in ligated-ileal loops

**Figure 13:**
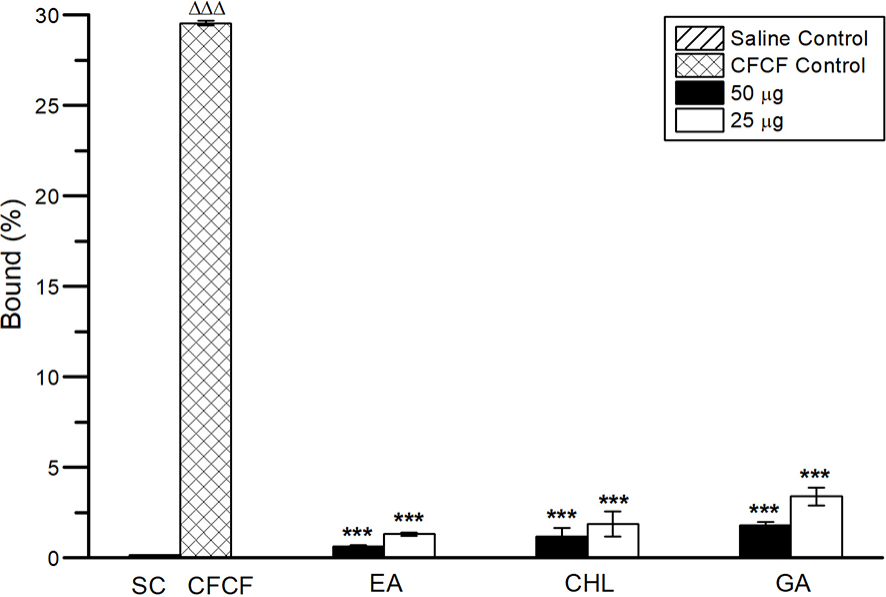
Cellular cAMP levels in ligated-ileal tissues. All values were expressed as mean ± SD, One Way Analysis of Variance (ANOVA) followed by the Dunnett test. *^ΔΔΔ^p*<0.001, compared with Saline control. ***p < 0.001 compared with CFCF control.

Cellular cAMP levels in ligated-ileal tissues were estimated as an increase in bound percent (B%) using competitive ELISA. A significant increase in cAMP was observed in CFCF control (*^ΔΔΔ^p*<0.001*)* compared with the saline control group. Three selected phenolic acids showed significant inhibitory activity (***p<0.001*)* against CFCF-induced elevation of cAMP levels in ileal tissues (**Figure 13**).

#### 3.3.3 Histopathology

**Figure 14:**
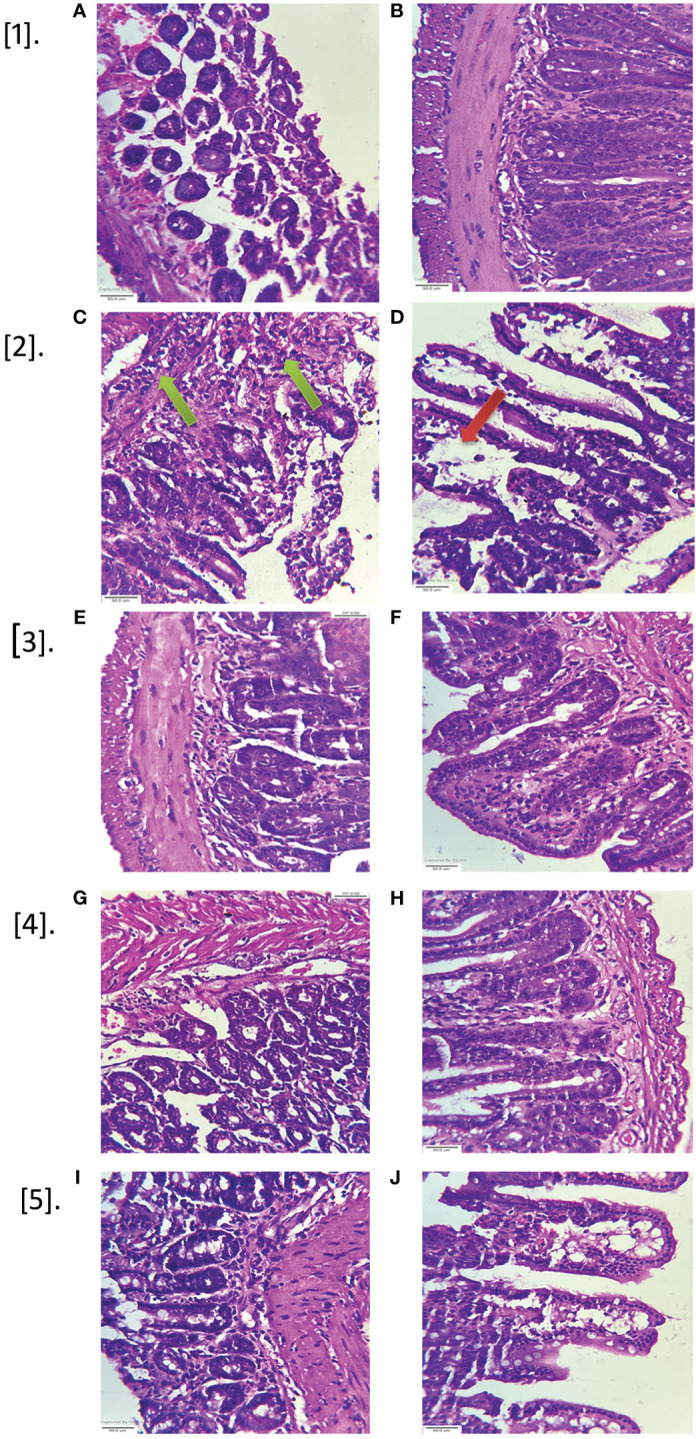
Histopathological analysis of mice ileal loops. Intestinal tissues of mice ileal sections stained with hematoxylin, eosin and observed at 40x magnification (scale bar=30µm). Green arrows represent inflammation in submucosa; red arrows represent mucosal layer degeneration and diffused edema. **[1A, B]** Saline control; **[2C, D]** CFCF control; **[3E, F]** EA+CFCF; **[4G, H]** CHL+CFCF; **[5I, J]** GA+CFCF.

The histological changes of ileal loop tissue sections of all groups were observed after staining with Eosin and Hematoxylin (**Figure 14**). After 18 hrs of treatment of CFCF, remarkable histopathological changes viz., increased inflammatory cellular infiltration in sub mucosa (green arrows, **Figure 14.2C**) along with degeneration of mucosal tissue and diffused edema in mucosal and sub mucosal layers (red arrows, **Figure 14.2D**) of the intestine were observed in CFCF control group compared to Saline control group (**Figures 14.2A, B**). Inflammation in sub mucosa is represented in **Figure 14.2C** and **14.2E. J, I** for CFCF control and test groups respectively whereas diffused edema and mucosal tissue degeneration in mucosa is shown in **Figure 14.2D** and **14.2F, H, J** for control and test groups. Among the pre-treated groups, 50ug of EA and CHL showed substantial protective activity against CFCF induced histopathological changes as evidenced by decreased inflammation and edema in the intestinal tissues (**Figures 14.2E, F** and **14.2G, H**). However, indistinct amelioration in histopathological changes was observed for the standard GA (**Figure 14.2I, J**) compared to CFCF control.

## 4 Discussion

Our current study investigated the anti-CT activity of formerly reported 20 polyphenolic compounds from three plants, i.e. *C. arborea*, *P. granatum*, and *P. guajava*, which are traditionally used to treat diarrheal diseases in India and were also reported to act against enteric pathogens. The bark of *C. arborea* significantly reduced castor oil-induced diarrhea in mice (Rahman et al., 2003). *P. guajava* leaf decoction has been shown to significantly reduce *Shigella flexener*i, enteropathogenic and enteroinvasive *Escherichia coli* (EPEC, EIEC) adherence to Hep-2 cells and to prevent CT and *E. coli* labile toxin binding to GM1 receptor (Birdi et al., 2010). Both the peel and juice extract of *P. granatum* showed gastroprotective activity and also prevented castor oil-induced enteropooling in rats (Souli et al., 2015; Zhao et al., 2018). Hydro-alcoholic extracts of *C*. *arborea* (bark) and *P*. *granatum* (peel) and *P*. *guajava* (leaf) significantly reduced CT-induced fluid accumulation, cellular cAMP levels and histopathological changes in ligated-ileal loops of adult mouse; *C. arborea* and *P*. *granatum* exhibited >90% binding inhibition of CT to GM1 receptor (Charla et al., 2022). Using molecular modelling studies, two phenolic acids (EA and CHL) and two flavonoids (PHD and RTN) were selected from these plants based on their binding affinity and interactions with active site amino acid residues involved in stable binding of CTB to the GM1 receptor on intestinal epithelial cells. Further, GA was selected as a standard for phenolic acids as it is reported to inhibit CT binding to GM1 receptor (Birdi et al., 2010) and QRTN as a flavonoid standard as it was reported to decrease CT-induced cAMP levels (Cherubin et al., 2016). The efficacy of these compounds against CT toxicity has been further corroborated by *in vitro* and *in vivo* assays.

The 3D structure of CT (1XTC) was retrieved from the RCSB structural database and missing residues in the structure were added. Among five β-subunits of CT (chain B, C, D, E, F), we have considered chain F as it formed a larger cavity at the GM1 binding interface, and the grid box was set around binding pocket residues that interacted with GM1 *viz.* Glu11, His13, Gly33, Glu51, Gln56, Gln61, Trp88, Asn90, and Lys91 (Merritt et al., 1994). Substitution of Gly33 residue with Asp was reported to abolish the binding affinity of CTB to GM1 (Merritt et al., 1995). From the selected 20 compounds, EA, CHL, Delphinidin, and Procyanidin B3 showed H-bond interactions with Gly33. The maximum number of interactions with active site residues was observed for Ellagic acid (10), followed by Procyanidin and Delphinidin. Based on drug-likeness score, grid score, and a total number of interactions, CT-EA (-36.42, 10), CT-CHL (-39.26, 9), CT-RTN (-48.02, 6), and CT-PHD (-44.39, 6) complexes were further analyzed for their stability by 100ns MD simulations along with their standards, CT-GA (-28.35, 7) and CT-QRTN (-37.25, 3).

Simulation studies using gromacs were performed for ligand-CT complexes (CT-EA, CT-CHL, CT-GA, CT-RTN, CT-PHD, CT-QRTN), and their structural stability was studied by analysing the root mean square deviation (RMSD), root mean square fluctuation (RMSF), radius of gyration (Rg), and solvent accessible surface area (SASA). The complexes CT-RTN, CT-EA, and CT-PHD showed relatively stable dynamics and reached an equilibrium state earlier within 35ns, of which the flavonoid standard QRTN showed the least RMSD value amongst all the studied complexes (**Figures 4A, B**). RMSF analysis over the C-α atoms of the chain having binding pocket showed a similar trend in the residual fluctuations in all the complexes. Also, it is interesting to observe that phenolic acid complexes (CT-EA and CT-CHL) showed the least RMSF values compared to the GA standard. However, flavonoid (CT-PHD) showed relatively less fluctuation than the standard (CT-QRTN) (**Figure 4C**). The R*g* values for complex CT-EA and CT-PHD showed much more stable R*g* values after the equilibration period of 40 ns. The CT-QRTN complex also showed a stable R*g*value comparable to the CT-PHD (**Figures 5A, B**). The complexes CT-EA, CT-PHD, and CT-GA showed stable dynamics through the conserved binding pocket interactions as observed in the MD simulation movies (**Supplementary Videos 1-6**). During the MD simulation, EA and PHD fit well in the binding pocket, while RTN moves away from the binding pocket after 30 ns. The complex CT-GA chain-F showed an increased SASA value, highlighting the exposure of the chain F to the solvent (**Figures 5C, D**). Complex CT-PHD and CT-EA showed large variations in the SASA value in chain F and it further decreased, hence making these complexes very stable during the course of simulation of 100 ns.

CT-PHD shows the maximum number of H-bonding interactions (8) formed during MD simulation; these interactions are consistent throughout the simulation period, whereas the number of H-bonds decreased from 5 to 2 for CT-RTN. CT-QRTN also shows a decrease in the H-bonding interactions during simulation. In general, flavonoids complexed with CT show a relatively higher number of H-bonds than phenolic acids. CT-EA complex initially showed ~7 H-bonding interactions, but steadily these interactions decreased to 3, which were consistent throughout the simulation. We also analyzed other non-bonded contacts of six compounds with CT. This shows that in complexes CT-PHD and CT-EA, the number of contacts steadily increases, making these complexes very stable. However, in other complexes, the number of contacts is either decreasing or steady but less in number. It is interesting to note that flavonoids and phenolic acids selected have maximum contact when compared to their respective standards. Analysis of secondary structural changes of six complexes over the simulation period revealed higher stability for CT-EA and CT-PHD complexes. Calculations of residue decomposition energy showed negative contribution energy by most of the residues in CT-EA, CT-CHL, and CT-PHD, favoring their stable complex formation. From the cumulative analysis of results from MD simulations, it is evident that CT-EA and CT-PHD showed stable dynamics compared with standards CT-GA and CT-QRTN. The order of stability of six complexes as observed through 100ns MD simulations and binding free energy were ranked as CT-EA > CT-CHL > CT-GA for phenolic acids and CT-PHD > CT-QRTN > CT-RTN for flavonoids. Subsequently, the efficacy of six complexes against CT binding to ganglioside GM1 was further validated experimentally by *in vitro* assays.

Both the qualitative and quantitative analysis of phenolic acids and flavonoids selected using *in silico* studies was performed HPTLC. The phytochemical analysis showed the presence of phenolic acids, EA and CHL in all three plants; GA in *C. arborea* and *P. guajava* whereas flavonoids PHD, RTN and QRTN in *P. guajava* and *P. granatum* in varying concentrations (**Table 3**). EA, an organic heterotetracyclic compound formed by dimerization of gallic acid, is the most active and abundant phytocompound found in *P. granatum* (Usta et al., 2013). It is an investigational drug and reported to show several therapeutic properties as an anti-oxidant and anti-proliferative agent (Losso et al., 2004; Han et al., 2006). CHL, an ester formed by the condensation of caffeic acid and quinic acid, is found majorly in coffee and black tea (Farah et al., 2008). It is reported to activate the immune system and also act as a potent anti-oxidant and carcinogenic inhibitor (Sato et al., 2011; Wang et al., 2020). RTN prevents displacing of reduced CTA1 from CT holotoxin in the endoplasmic reticulum of the host cell by inducing conformational changes in protein disulfide isomerize (PDI), thus restricting the translocation of CTA1 into the cytosol as reported (Guyette et al., 2019).

Cell free culture filtrate of O1 El Tor Ogawa *V*. *cholerae* isolated from clinical samples during the cholera out-breaks in Karnataka, India (Roy et al., 2012; Roy et al., 2014) was utilized for both *in vitro* as well as *in vivo* studies. It was reported in earlier studies that CT is secreted by the bacterium actively in AK1 media (Iwanaga et al., 1986). The clinical strain of *V. cholerae* used in the current study efficiently secreted CT into CFCF when grown in AK1 media, and the concentration of CT was determined as 19.78 ± 0.52μg/mL using GM1 ELISA. MTT cytotoxicity assay was performed with six compounds on the CHO cell line to determine their non-cytotoxic doses that showed > 90% cell viability, and further *in vitro* assays were carried out using their respective NC1, NC2. The neutralizing activity of selected phenolic acids and flavonoids against CT (1600ng/mL) binding to the GM1 receptor was evaluated as BI% using the GM1 ELISA. Phenolic acids EA (97%, 97%) and CHL (92%, 81%) demonstrated remarkable BI% at NC1 and NC2 when compared to their standard GA (31%, 12%). Among flavonoids, PHD (59%, 24%) showed prominent BI% compared with RTN (9%, 2%) and standard QRTN (23%, 6%) at NC1 and NC2 respectively. Earlier similar studies with GA (50mM) showed 40% binding inhibition of CT to GM1 (Birdi et al., 2010) and in another study, binding of FITC-CTB to Vero cells in the presence of 10µg/ml GA and QRTN was inhibited by 23% and 20%, respectively (Cherubin et al., 2016). These results explain that the interaction of phenolic acids EA and CHL with β-pentamer efficiently inhibits CT binding to GM1 receptor and eventually neutralizes CT toxicity by preventing its internalization into enterocytes. Further confirmatory studies were performed to investigate the protective activity of six compounds against CT-induced cell elongation and elevation of cAMP levels in the CHO cell line.

The hallmark of CT toxicity is an increase in cellular cAMP levels that results in the dramatic efflux of ions and water from infected enterocytes, leading to diarrhea (Iwaz et al., 1986). The protective activity of six compounds against CT-induced elevation of cAMP in CHO cell line was evaluated using competitive cAMP ELISA as an increase in bound percent (B%) of cAMP. Phenolic acids showed a significant increase (*p<*0.001) in B%, i.e. EA (82%, 81%), CHL (81%, 78%) and GA (80%, 60%) at NC1 and NC2 respectively compared with CFCF control (44%). Flavonoids showed the least protective activity compared to phenolic acids, i.e. PHD (71%, 50%), RTN (53%, 44%), and QRTN (66%, 47%) at NC1 and NC2 respectively. Surprisingly, EA, CHL, and PHD showed efficient defensive activity against CT-induced cAMP levels compared to standard GA and QRTN. Similar results were observed in an earlier study that showed 76% and 59% of decrease in cAMP with 10µg/mL of GA and QRTN compared to CT control (Cherubin et al., 2016). However, no previous reports were found for the remaining four compounds. The results from the cAMP assay were correlated with the protective activity of six compounds against CT-induced cell elongation in the CHO cell line. The protection against CT-induced cell elongation in CHO cells was observed to be prominent in the presence of EA and CHL, which were consistent with the results from the cAMP assay (**Figures 11.5I–6L**).

From the cumulative results of *in silico* and *in vitro* studies, it is apparent that there is a differential activity of phenolic acids and flavonoids against CT. From docking and simulation analysis, it is evident that EA, CHL and PHD formed more stable complexes with CT. However, phenolic acids EA and CHL were found to be effective in inhibiting CT binding to the GM1 receptor and also significantly reduced CT-induced cAMP levels and cell elongation in CHO cell lines *in vitro*. When MD simulation analysis of six complexes was compared with *in vitro* results, EA, CHL and PHD were observed to form stable complexes with CT during simulations as well as inhibit CT binding to GM1 and further CT toxicities. From the studied six complexes, EA and CHL that showed distinguished activity against CT *in vitro* were further validated *in vivo* against CT-induced fluid accumulation using adult mice ligated-ileal loop model along with standard GA.

The enterotoxigenic activity of CT causes fluid accumulation in the ligated-ileal loops of adult mice. CFCF containing 1μg of CT when instilled into distal ligated ileal loops of 2cm in length, elicited fluid accumulation after 18hrs and loop mean (n=6) weight/length ratio was estimated 0.25g/cm ± 0.023 whereas the similar study by Sawasvirojwong et al. (2013) showed 0.192 ± 0.042g/cm. This fluid accumulation induced by CT was observed to be significant (*p*<0.001*)* compared with the Saline control group (0.09 ± 0.1g/cm). Pre-incubated mixture of CFCF containing 1µg of CT and EA/CHL at two different concentrations (50µg, 25µg) showed a significant reduction (*p*<0.001) in CT-induced fluid accumulation. GA showed a significant decrease (*p*<0.01) against CT-induced fluid accumulation at a higher concentration but was found to be less effective at a lower concentration (25µg). Cellular cAMP levels in ligated-ileal tissues were estimated as an increase in bound percent (B%) of cAMP using competitive ELISA. Significant increase in cAMP levels *(p*<0.001*)* were observed in the CFCF control group (29.5%) compared to the Saline control (0.1%). EA (0.62%, 1.31%), CHL (1.18%, 1.89%), and GA (1.79%, 3.39%) showed significant reduction in cAMP levels (*p*<0.001) at 50µgand 25µg respectively. Histopathological analysis of CFCF treated ileal loops showed inflammation in sub mucosa and diffused edema in the mucosal layer of the intestine and similar results were also reported previously (Sawasvirojwong et al., 2013). CFCF treated ileal loops in the presence of EA and CHL showed a prominent reduction in inflammation and edema. However, with GA, amelioration of histopathological was found to a lesser extent compared with CFCF control.

Polyphenols were reported to show anti-CT activity either by inhibiting CTB binding to GM1 receptor thus blocking CT entry into host cells or by interfering with CTA1 induced catalysis of ADP-ribosylation of Gsα that results in continuous stimulation of adenylate cyclase and increased cellular cAMP levels. Tea catechins and 6-gingerol have been shown to provide resistance to *V. cholerae*-induced fluid accumulation in rabbit ligated intestinal loops by blocking CT binding to GM1 and preventing its internalisation into enterocytes (Toda et al., 1992; Saha et al., 2013). Apple and grape polyphenols were also reported to inhibit CT toxicity by various mechanisms, which included their interference with CTA1 catalytic activity and CTB binding to GM1 receptor (Saito et al., 2002; Cherubin et al., 2016). These leads from the literature encouraged us to further investigate the role of polyphenols in preventing CT-induced diarrhea.

## 5 Conclusion

The present study investigated the inhibitory activity of polyphenolic compounds from *C. arborea, P. granatum*, and *P. guajava* against CT. Based on computational screening, two flavonoids and two phenolic acids were selected along with reported known inhibitiors for further experimental validation. Among the selected six compounds, EA and CHL showed efficient inhibitory activity against CT-induced toxicities *in vitro*. Further, *in vivo* studies with EA and CHL were correlated with *in vitro* results as evidenced by decreased CT-induced fluid accumulation and histopathological changes in the adult mouse ligated-ileal loop model. Thus, our observations from computational molecular modelling studies are in good agreement with experimental results. Our study elucidated the inhibitory activity of EA and CHL against CT by interacting with B-pentamer and subsequently inhibiting CT binding to the GM1 receptor on enterocytes. Clinical studies would further help to validate the application of EA and CHL as a supplement to standard therapies during cholera outbreaks.

## Data availability statement

The original contributions presented in the study are included in the article/**Supplementary Material**. Further inquiries can be directed to the corresponding authors.

## Ethics statement

The animal study was reviewed and approved by ICMR-NITM, Belagavi (Accession number: IAEC/ICMR-NITM BGM/2018/07).

## Author contributions

RC performed *in vitro*, *in vivo*, and *in silico* studies, conducted a literature review, and wrote the original draft. PPP and VSP assisted RC in experimentation, reviewed and edited the manuscript. VVB and VK assisted RC in executing computational studies. VB assisted in performing ileal-ligation in adult mouse. RJ assisted in performing HPTLC analysis and revised the manuscript. DH analyzed the findings, revised and edited the manuscript. SR designed the study, oversaw its execution, revised and finalized the manuscript. All authors contributed to the article and approved the submitted version.
