# Supplementary material for: Anti-Cholera toxin activity of selected polyphenols from Careya arborea, Punica granatum, and Psidium guajava
Source: Front Cell Infect Microbiol. 2023 Apr 11;13:1106293. doi: 10.3389/fcimb.2023.1106293 (PMC10126245; doi:10.3389/fcimb.2023.1106293)

**Supplementary data:**

**Supplementary Table 1** List of 20 polyphenolic compounds selected from C. arborea, P. granatum, and P. guajava using databases and literature survey.

| S. No. | Plant name | Name of the compound | PubChem ID | Classification |
| --- | --- | --- | --- | --- |
| 1. | *Punica granatum* | Catechin | 9064 | Flavonoid |
| 2. | *Psidium guajava, Careya arborea, Punica granatum* | Quercetin | 5280343 | Flavonoid |
| 3 | *Psidium guajava, Careya arborea, Punica granatum* | Ellagic acid | 5281855 | Phenolic acid |
| 4 | *Punica granatum* | Chlorogenic acid | 1794427 | Phenolic acid |
| 5 | *Punica granatum* | Kaempferol | 5280863 | Flavonoid |
| 6 | *Punica granatum, Psidium guajava* | Rutin | 5280805 | Flavonoid |
| 7 | *Punica granatum* | Luteolin | 5280445 | Flavonoid |
| 8 | *Punica granatum* | Cyanidin | 128861 | Flavanoid |
| 9 | *Psidium guajava, Punica granatum* | Gallic acid | 370 | Phenolic acid |
| 10 | *Punica granatum* | Phlorizin | 6072 | Flavonoid |
| 11 | *Punica granatum* | Caffeic acid | 689043 | Phenolic acid |
| 12 | *Punica granatum* | Phellatin | 44258781 | Flavonoid |
| 13 | *Punica granatum* | Delphinidin 3-glucoside | 443640 | Flavonoid |
| 14 | *Punica granatum* | Ferulic acid | 445858 | Phenolic acid |
| 15 | *Psidium guajava* | Gentisic acid | 3469 | Phenolic acid |
| 16 | *Psidium guajava* | Procyanidin b1 | 11250133 | Flavonoid |
| 17 | *Psidium guajava* | Procyanidin b2 | 122738 | Flavonoid |
| 18 | *Psidium guajava* | Procyanidin b3 | 146798 | Flavonoid |
| 19 | *Psidium guajava* | Quercitrin | 5280459 | Flavonoid |
| 20 | *Psidium guajava* | Eugenol | 3314 | Phenolic acid |

**Supplementary Table 2** The grid score resulted from flexible virtual screening and other energy components van der Waals (vdw), electrostatic energy (es) of 13 flavonoids docked with 1XTC (Chain F).

| Pubchem CID | Compound name | Grid_Score (kcal/mol) | Grid_vdw_energy (kcal/mol) | Grid_es_energy (kcal/mol) |
| --- | --- | --- | --- | --- |
| 5280805 | Rutin | -48.042801 | -45.469723 | -2.573079 |
| 6072 | Phlorizin | -44.387592 | -40.547153 | -3.840439 |
| 44258781 | Phellatin | -43.699451 | -40.455997 | -3.243455 |
| 122738 | Procyanidin b2 | -43.479301 | -40.645905 | -2.833396 |
| 11250133 | Procyanidin b1 | -42.414948 | -39.893745 | -2.521202 |
| 146798 | Procyanidin b3 | -40.048206 | -39.379646 | -0.668559 |
| 443650 | Delphinidin | -38.179119 | -32.950298 | -5.22882 |
| 5280459 | Quercetrin | -37.249252 | -34.549435 | -2.699819 |
| 5280343 | Quercetin | -35.541042 | -31.458048 | -4.082994 |
| 5280863 | Kaempferol | -34.439274 | -30.487968 | -3.951307 |
| 5280445 | Luteolin | -33.997169 | -29.748465 | -4.248707 |
| 128861 | Cyanidin | -32.527771 | -27.560312 | -4.967459 |
| 9064 | Catechin | -31.774496 | -28.145555 | -3.628941 |

**Supplementary Table 3** The grid score resulted from flexible virtual screening and other energy components van der Waals (vdw), electrostatic energy (es) of 7 phenolic acids docked with 1XTC (Chain F).

| Pubchem ID | Compound name | Grid_Score | Grid_vdw_energy | Grid_es_energy |
| --- | --- | --- | --- | --- |
| 1794427 | Chlorogenic acid | -39.264503 | -34.880714 | -4.383787 |
| 5281855 | Ellagic acid | -36.419472 | -31.110126 | -5.309345 |
| 689043 | Caffiec acid | -28.51786 | -23.899292 | -4.618569 |
| 370 | Gallic acid | -28.355522 | -23.503485 | -4.852037 |
| 445858 | Ferulic acid | -27.67881 | -22.11463 | -4.24631 |
| 3469 | Gentistic acid | -26.730343 | -22.38484 | -4.345502 |
| 3314 | Eugenol | -25.38118 | -22.887379 | -2.4938 |

**Supplementary** **Table 4** ADMET profile and drug-likeness of top 10 compounds:

| Compound name | LogS | LogD | LogP | ^1^HIA | Caco-2 | ^2^PPB (%) | ^3^NPL | Ames | Carcinogenicity | Lipinski rule | GSK rule |
| --- | --- | --- | --- | --- | --- | --- | --- | --- | --- | --- | --- |
| Rutin (Flavonoid) | -3.93 | 0.69 | -0.76 | 0.92 | -6.34 | 83.81 | 2.01 | 0.80 | 0.06 | Rejected | Rejected |
| Phlorizin (Flavonoid) | -3.07 | 0.94 | -0.26 | 0.95 | -6.32 | 65.30 | 1.80 | 0.52 | 0.24 | Accepted | Rejected |
| Phellatin (Flavonoid) | -3.67 | 0.95 | 0.58 | 0.82 | -6.17 | 89.11 | 2.06 | 0.77 | 0.05 | Rejected | Rejected |
| Procyanidin (Flavonoid) | -3.96 | 1.66 | 1.85 | 0.96 | -6.77 | 88.71 | 1.93 | 0.27 | 0.03 | Rejected | Rejected |
| Delphinidin3-glucoside (Flavonoid) | -3.09 | 0.59 | -0.20 | 0.87 | -6.42 | 85.93 | 2.08 | 0.68 | 0.04 | Rejected | Rejected |
| Quercetrin (Flavonoid) | -4.03 | 1.59 | 0.82 | 0.53 | -6.14 | 89.52 | 2.16 | 0.82 | 0.07 | Rejected | Rejected |
| Chlorogenic acid (Phenolic acid) | -1.19 | 0.01 | -0.16 | 0.87 | -6.13 | 67.19 | 2.25 | 0.03 | 0.06 | Accepted | Accepted |
| Ellagic acid (Phenolic acid) | -4.60 | 0.79 | 1.12 | 0.198 | -5.31 | 78.23 | 0.59 | 0.38 | 0.31 | Accepted | Accepted |
| Gallic acid (Phenolicacid) | -1.2 | 0.34 | 0.64 | 0.08 | -5.73 | 53.49 | 0.98 | 0.05 | 0.02 | Accepted | Accepted |

^1^Human intestinal absorption; ^2^Plasma protein binding; ^3^Natural product likeness

**Supplementary Table 5** List of non-bonded interactions in six complexes before and after simulations

| Compound name | Interactions of herbal compounds with 1XTC at 0 nano seconds (Before simulation studies) | | | | | Interactions of herbal compounds with 1XTC at 100 nano seconds (After simulation studies) | | | | |
| --- | --- | --- | --- | --- | --- | --- | --- | --- | --- | --- |
|  | Hydrogen bond interactions (No. of interactions | Van der Waals, Pi-Alkyl, CH, Pi-Cation, Pi-Sigma, Pi-Pi stacked, Pi-Pi T-shaped interactions (No. of interactions) | Active site residues within interactions | Number of interactions with active site residues | Total no. Of interactions | Hydrogen bond interactions (No. of interactions | Van der Waals, Pi-Alkyl, CH, Pi-Cation, Pi-Sigma, Pi-Pi stacked, Pi-Pi T-shaped interactions (No. of interactions) | Active site residues within interactions | Number of interactions with active site residues | Total no. Of interactions |
| Rutin  (Flavonoid | Asn14, GLN61, Glu51, Gln56(2) | His13 | Asn14, GLN61, Glu51, Gln56, His13 | 6 | 6 | Asn44 [2] | Thr19, Ala46 [2] | Nil | Nil | 4 |
| Phlorizin  (Flavonoid) | Asn90, Asn14, Glu51 | His57, Trp88(2), Ile58 | Asn90, Asn14, Glu51 Trp88,Ile58 | 6 | 7 | Lys91 [3], Val52 [3], Glu51 [2] | Trp88 [1] | Lys91, Glu51, Trp88 | 6 | 9 |
| Quercetrin  (Flavonoid) | Asn14, Gln61, Gln56 | His57, Trp88 | Gln61, Gln56, Trp88 | 3 | 5 | Gln61 [4], Gln56, Leu31, Ser60, Glu36 | Lys34 [3], Ile58 | Gln61, Gln56 | 5 | 12 |
| Chlorogenic acid  (Phenolic acid) | Gly33, Gln61, Trp88, Lys91, Gln56(3), Gln61 | Nil | Gly33, Gln61, Trp88, Lys91, Gln56, Gln61 | 8 | 8 | Asp7 | Nil | Nil | Nil | 1 |
| Ellagic acid  (Phenolic acid) | Gln61(2), Asn90(2), Glu51, Gly33, His57 | Trp88(5) | Gln61, Asn90, Glu51, Gly33, Trp88 | 10 | 12 | Glu51 | Trp88 [7], Ala95 | Trp88, Glu51 | 8 | 9 |
| Gallic acid  (Phenolic acid | Gln61(2), Asn90(2), Glu51, His57 | Trp88(2) | Gln61, Asn90, Glu51, Trp88 | 7 | 6 | Glu51 [2] | Trp88 [2] | Glu51, Trp88 | 4 | 4 |

**Supplementary Table 6** The mean W/L ratio of control and tested groups using adult mice ligated-ileal loop assay

| Group name | Mean W/L ratio | SE |
| --- | --- | --- |
| Saline control (100µl) | 0.078 | 0.011 |
| CFCF control (CT=1µg ) | 0.249 | 0.023 |
| EA (50µg) | 0.091 | 0.005 |
| EA (25µg) | 0.094 | 0.010 |
| CHL (50µg) | 0.132 | 0.014 |
| CHL (25µg) | 0.176 | 0.016 |
| GA (50µg) | 0.188 | 0.021 |
| GA (25µg) | 0.222 | 0.009 |

**Supplementary Figure 1.** Temperature and pressure equilibration plot of six complexes.


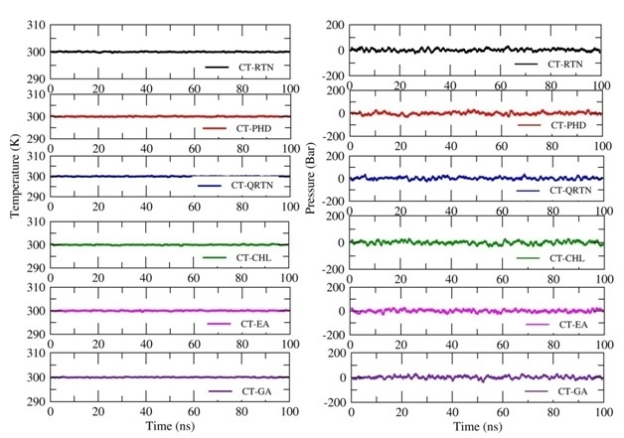


**Supplementary Figure 2.** The maximum number of H-bonds promoting stable complex formation for all the CT-complexes are shown in A and number of H-bonding interactions between CT chain F are shown in B. The number of non-bonded contacts including hydrophobic and electrostatic interactions observed between the pentameric CT subunits and ligands are shown in C for all the complexes.


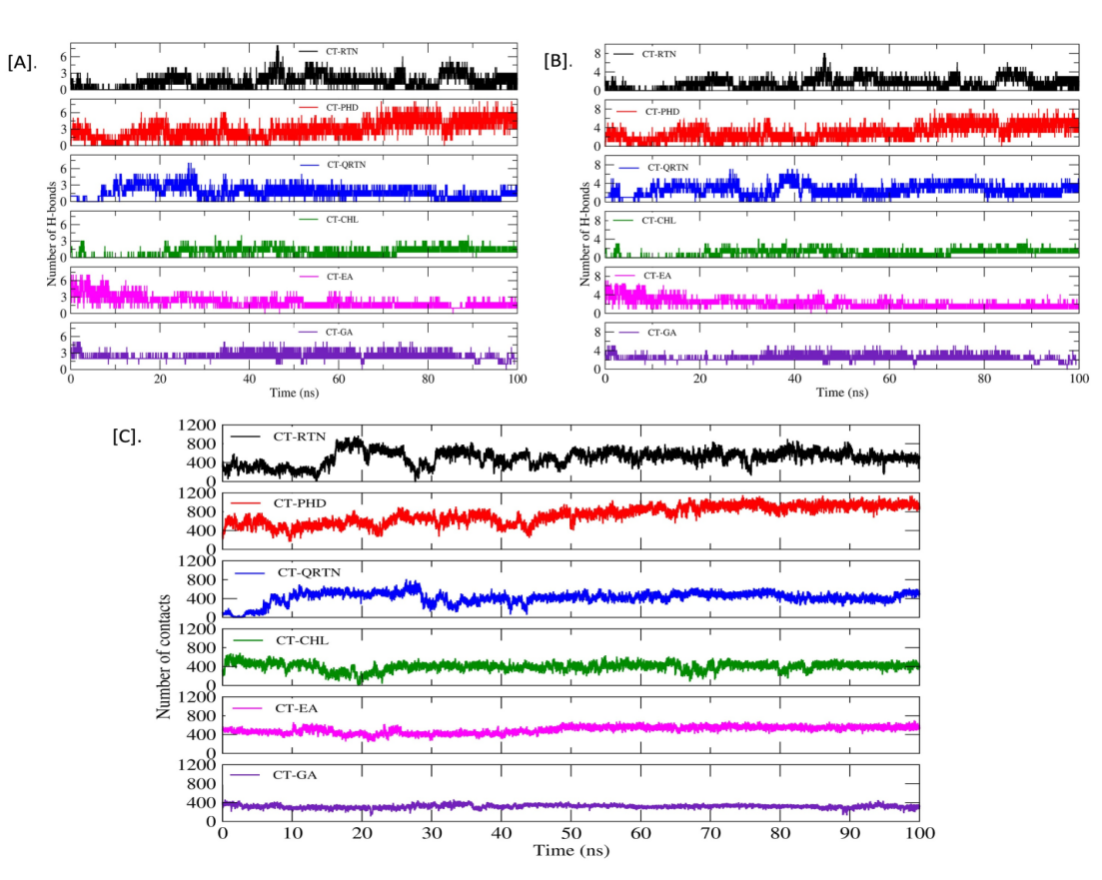


**Supplementary Figure 3:** The distortion in the secondary structures during the 100ns MD simulation for complexes CT-RTN[A], CT-PHD[B], CT-QRTN[C], CT-CHL[D], CT-EA[E], CT-GA[F].


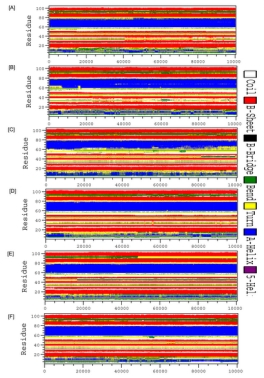


**Supplementary Figure 4.** Per residue decomposition energy reveals the contribution of key binding site residues in the binding energy.


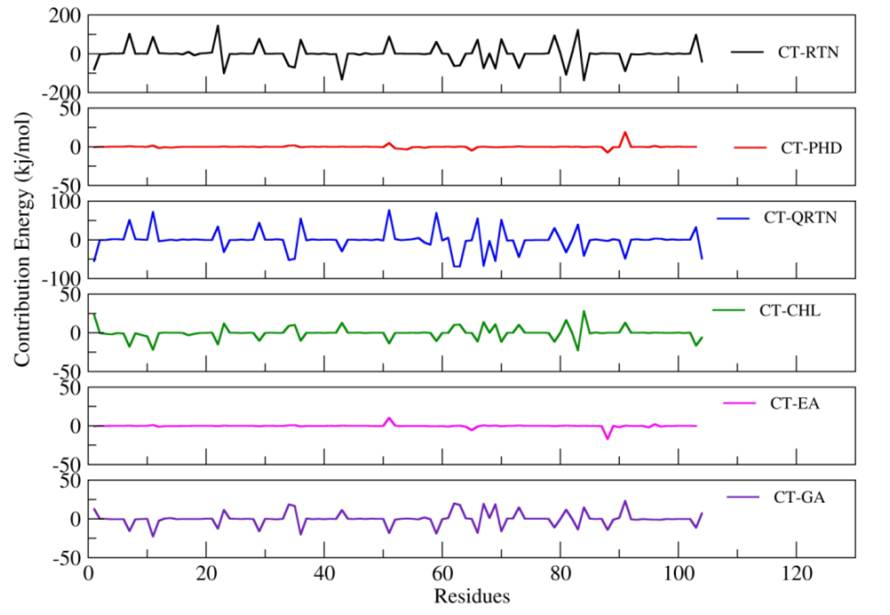


**Supplementary Figure 5.** HPTLC-PDA chromatogram of phenolics (a) at 280nmand flavonoids (b) at 310nm in *C. arborea,* *P. guajava* and *P. granatum*


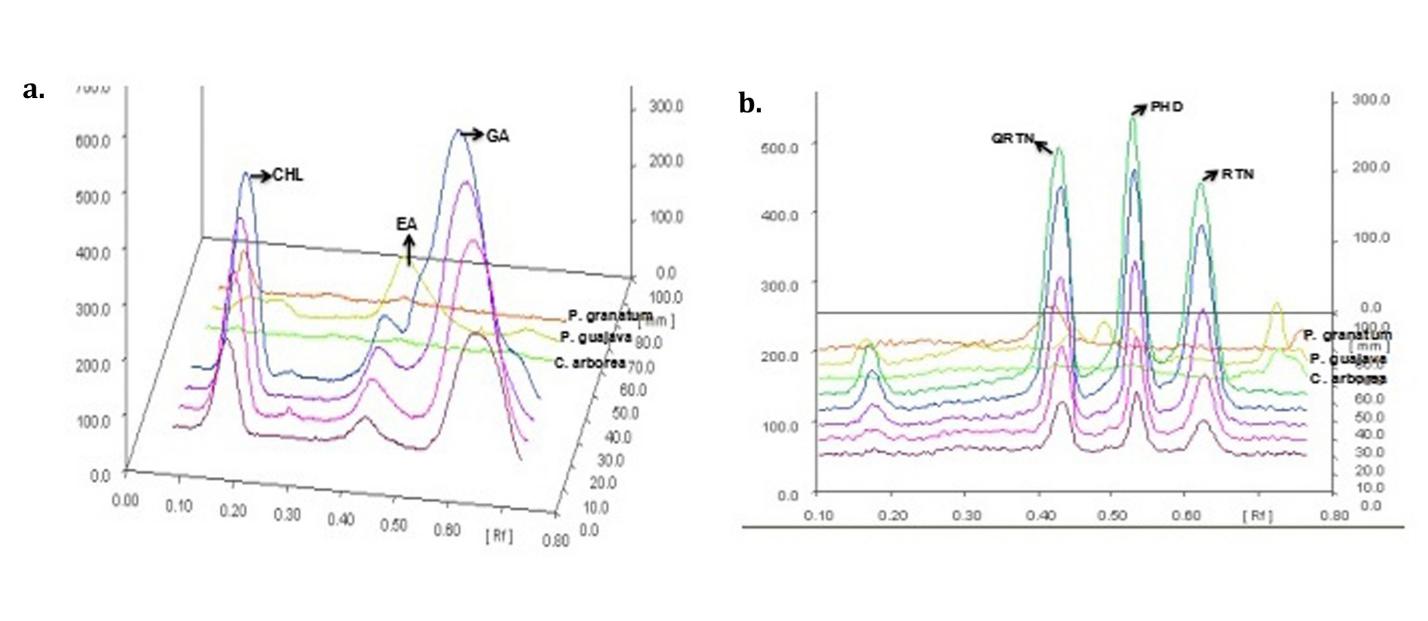

Supplement: Supplementary file 7 [file DataSheet_1.docx]
